# Integration of single-cell sequencing, transcriptome sequencing, and machine learning for constructing and validating histone acetylation-related prognostic risk models in hepatocellular carcinoma

**DOI:** 10.3389/fimmu.2026.1624883

**Published:** 2026-01-23

**Authors:** Yajie Qi, Fulin Wang, Wenchao Ren, Chuanxu Cai, Yichen Zhou, Pengpeng Zhu, Puyi He, Qian Wang

**Affiliations:** 1 National and Local Joint Engineering Research Center of Biodiagnosis and Biotherapy, The Second Affiliated Hospital of Xi’an Jiaotong University, Xi’an, China; 2 Tumor and Immunology Center of Precision Medicine Institute, Xi’an Jiaotong University, Xi’an, China; 3 General Surgery, The Second Affiliated Hospital of Dalian Medical University, Dalian, China; 4 General Surgery, The Second Hospital of Lanzhou University, Lanzhou, China

**Keywords:** histone acetylation, immune microenvironment, LIHC, machine learning, NEU1

## Abstract

**Background:**

Liver hepatocellular carcinoma (LIHC), a prevalent gastrointestinal malignancy, continues to demonstrate poor prognosis despite therapeutic advances improving clinical outcomes. Histone acetylation, a key epigenetic modification, regulates critical processes including chromatin remodeling, gene expression and drives tumor progression in multiple cancers (e.g., lung, gastric) yet its systemic role in LIHC remains unclear.

**Methods:**

This study integrated LIHC single-cell/RNA-seq data and histone acetylation-related gene sets to construct a LIHC risk prediction model based on histone acetylation-related genes using 101 machine learning combinatorial algorithms. The model’s comprehensive value was evaluated through prognostic analysis, pathway enrichment analysis, immune landscape analysis, chemosensitivity analysis, mutation analysis, ferroptosis, and m6A methylation analysis. NEU1’s functional role was investigated via cell communication networks and molecular docking. Experimental validation included *in vitro* assays (Cell Counting Kit-8, migration, invasion), and clinical sample verification (quantitative real-time PCR (qRT-PCR) and Western Blot (WB)); these were performed to validate the key findings.

**Results:**

Using 101 machine learning combinations, we constructed an 11-gene LIHC risk model (HLA-B, HEXB, CDK4, ACAT1, NAA10, B2M, HSPD1, NPM1, PON1, NEU1, CFB) demonstrating robust prognostic accuracy across training/validation cohorts and 10 LIHC subtypes. Immune landscape analysis revealed that the high-risk group exhibited higher tumor purity and lower immune infiltration, with better responses to PD-L1 and PD-L2 treatment. Chemosensitivity analysis showed that the high-risk group had increased sensitivity to four drugs, including Axitinib, but decreased sensitivity to 21 drugs, including Cisplatin. The risk model score significantly correlated with the expression levels of ferroptosis-related genes such as GPX4 and m6A methylation-related genes such as METTL3. NEU1 was identified as a key risk factor in this model, with the NEU1 high-expression group showing of intercellular communication in endothelial cells and other cell types. Pseudotime analysis suggested that NEU1 may promote LIHC progression by blocking normal differentiation of endothelial cells. Molecular docking revealed that five compounds, including Oseltamivir, could bind directly to NEU1. Knockdown of NEU1 significantly reduced proliferation, migration, and invasion of LIHC cells, and slowed LIHC tumor growth.

**Conclusions:**

We constructed a histone acetylation-based risk model for LIHC diagnosis, prognosis, and therapy, identifying NEU1 as a key biomarker and potential therapeutic target.

## 1 Introduction

Primary liver cancer is the sixth most common cancer and the third leading cause of cancer-related mortality worldwide, with hepatocellular carcinoma (LIHC) accounting for 75–90% of cases. It is characterized by complex etiology, insidious early symptoms, and limited therapeutic options, which imposes a substantial burden on global healthcare systems (1–3). Current LIHC risk stratification relies heavily on clinical staging (e.g., BCLC system) and genomic features (e.g., TP53 mutations), yet these models exhibit limited accuracy in predicting immunotherapy response and chemosensitivity (4, 5).

Recent advances in omics technologies—particularly single-cell RNA sequencing (scRNA-seq) and bulk transcriptome sequencing—have revolutionized cancer research. scRNA-seq resolves cellular heterogeneity within the tumor microenvironment with unprecedented resolution, enabling identification of rare cell populations and dysregulated intercellular communication networks (6–8). Bulk RNA-seq, in contrast, provides a holistic view of transcriptomic alterations driving tumor progression (9). Despite their utility, few LIHC studies have integrated these approaches with epigenetic mechanisms such as histone acetylation.

Histone Acetylation (HAA) is an important chemical modification in epigenetics, that regulates can regulate the expression of relevant genes by affecting chromatin remodeling, transcription factor activity, and metabolic enzyme activity. It thus plays an important role in biological processes such as embryonic development, immune cell activation, maintenance of hepatocyte function, DNA damage repair, and metabolic regulation (10–13). In recent years, numerous of studies have shown that histone acetylation plays an important role in the development of malignant tumors such as breast cancer and gliomas; for example, Kim JJ et al. found that in breast cancer, the *in vitro* replication ability of breast cancer cells was reduced after histone H4K8 was acetylated by KAT2B, and that the reduction in the level of KAT2B might enhance resistance to PARP inhibitors (14). In gliomas, Histone acetyltransferases (HATs) also promotes cancer progression by reprogramming the tumor immune microenvironment to suppress anti-tumor immune responses. Chen P et al. found that KAT13D is highly expressed in glioblastoma stem-like cells and promotes microglial cell infiltration and its polarization towards an immunosuppressive phenotype by regulating HIF1α signaling and the transcription of chemokines OLFML3 and LGMN, thereby promoting glioma progression (15). Guo X et al. showed that a risk model consisting of histone acetylation-related genes HDAC6, CREB3, KLF13, GOLGA2, RPS6KA1, and ZMIZ2 in acute myeloid leukemia exhibits strong diagnostic and prognostic predictive power (16). However, despite its established roles in other cancers, the systematic impact of histone acetylation-related genes on LIHC prognosis and therapy resistance remains poorly characterized. Given the limited therapeutic options and high mortality of LIHC, there is an urgent need to identify novel epigenetic biomarkers for risk stratification and targeted interventions. This study aims to bridge this gap by integrating multi-omics data to construct the first histone acetylation-based prognostic model for LIHC, thereby uncovering potential therapeutic targets (e.g., NEU1) and providing a foundation for precision oncology.

In this study, we integrated single-cell RNA sequencing data from LIHC, RNA sequencing data, and histone acetylation-related gene sets to construct a model containing 11 histone acetylation-related genes (HLA-B, HEXB, CDK4, ACAT1, NAA10, B2M, HSPD1, NPM1, PON1, NEU1, and CFB) using 101 machine learning algorithms to construct a prognostic risk model. We comprehensively evaluated the function of the model in terms of LIHC diagnosis, prognosis, potential pathways, immune infiltration, and chemotherapy, identified NEU1 as a key risk factor of the model, and further explored its value in cell communication and pseudotime analysis. In addition, we validated the expression of these 11 genes in clinical samples and the effect of NEU1 on the malignant behavior of LIHC cells. Our study suggests that the prognostic risk model based on histone acetylation-related genes has potential clinical applications in LIHC.

## 2 Materials and methods

### 2.1 Data acquisition and processing

The RNA sequencing data and corresponding clinical information for LIHC were obtained from the TCGA database (https://portal.gdc.cancer.gov/) (356 samples). Data for 212 samples from the International Cancer Genome Consortium Liver Cancer (ICGC-LIHC) cohort were sourced from the LIHCDB V2.0 database (http://lifeome.net:809/#/home), and data for another 206 samples from an ICGC-LIHC cohort were downloaded from the GEO database (https://www.ncbi.nlm.nih.gov/geo/). Specifically, the dataset GSE124751, comprising 206 samples, was retrieved from GEO. The “limma” package was used to correct for batch effects between data sets. After excluding patients with incomplete survival data, a total of 781 tumor samples were retained for subsequent analysis (9).

To identify genes significantly associated with histone acetylation, we utilized the GeneCards database (https://www.genecards.org/), a comprehensive and integrative database of human genes. We performed a search using the keyword “Histone Acetylation.” GeneCards calculates a Relevance Score for each gene, representing the strength of the association between the gene and the search term based on weighted evidence from diverse sources, including experimental data, genetic pathways, and literature text mining. To ensure the reliability and specificity of the retrieved gene set, we applied a filtering threshold to exclude genes with weak associations. Only genes with a Relevance Score greater than 30 were retained. This rigorous screening process resulted in a final set of 588 histone acetylation-related genes for subsequent analysis.

The full single-cell RNA sequencing (scRNA-seq) datasets GSE149614 (6) and GSE166635 (7) were acquired in the GEO database. The Seurat package (8) was used for quality control and downstream analysis of scRNA-seq data. We excluded cells expressing fewer than 200 genes and genes detected in fewer than 5 cells and retained cells with a gene count between 300 and 5000. To ensure adequate sequencing depth while removing technical outliers, the total RNA count per cell (nCount_RNA) was kept above 1000 but below the 97th percentile of the dataset’s count distribution. The PercentageFeatureSet function was used to calculate the percentage of mitochondrial gene expression in each cell, excluding cells with >20% mitochondrial gene expression or >5% hemoglobin gene expression. The final curated dataset contains 86,409 cells in 23 samples.

The “RunHarmony” function was used to correct batch effects in the scRNA-seq data. The optimal number of principal components (PCs) for downstream analysis was determined by the “ElbowPlot” function. Cell clustering was performed using the “FindClusters” function, followed by dimensionality reduction visualization using “RunUMAP” (dims=1:10). Cell populations were manually annotated based on classical cell type markers reported in the literature.

### 2.2 Sources of patient and tissue samples

Frozen tissues and corresponding non-tumor tissues from five patients diagnosed with liver malignancies were collected from the Second Affiliated Hospital of Xi’an Jiaotong University. All patients had a pathological diagnosis of hepatocellular carcinoma (LIHC) after surgical resection. Samples were used for WB and qRT-PCR experiments. All participants provided informed consent before the commencement of the study, which received approval from the Ethics Committee of the Second Affiliated Hospital of Xi’an Jiaotong University.

### 2.3 Cell culture

MHCC-97H, Hep3B, and LO2 cells were purchased from the Cell Bank of the Chinese Academy of Sciences and cultured in Dulbecco’s modified Eagle’s medium (DMEM) supplemented with 10% fetal bovine serum (FBS), 100 U/ml penicillin, and 100 μg/ml streptomycin in a cell culture incubator at 37°C, 5% CO2.

### 2.4 Survival validation in independent cohorts

The Kaplan-Meier method was used to plot the overall survival (OS) and disease-free survival (DFS) curves, and the log-rank test was performed to compare the survival differences between the high-risk and low-risk groups (P < 0.05 was considered statistically significant). In the ICGC-LIRI-JP cohort, subgroup analyses were stratified by age (≤60 years/>60 years), gender, viral etiology (HBV/HCV), TNM stage (III/III-IV), and fibrosis stage (F0-F2/F3-F4). In the GSE124751 cohort, subgroups were stratified by age (≤60 years/>60 years) and tumor mutational burden (TMB, divided into high/low TMB groups using the median as the cutoff). The prognostic value of the risk model was validated in each subgroup. All survival analyses were performed using the “survival” package in R software (version 4.2.1), and Kaplan-Meier curves were plotted using the “survminer” package.

### 2.5 Disease risk modeling and validation

The “FindMarkers” function of the Seurat package (8) was applied to identify differentially expressed genes (DEGs) in cancer and normal tissues. Statistical significance was determined using the Wilcoxon test (threshold |logFC|>0.5 and corrected p-value <0.05, default for remaining parameters). The DEGs of cancer and normal tissue cells were intersected with histone acetylation-related genes to obtain 66 genes and further intersected with all genes of TCGA-LIHC, ICGC-LIHC, and GSE124751 tumor samples to obtain 65 genes, defined as histone acetylation-related differentially expressed genes (HAc-related DEGs).

To build a robust prognostic signature, we conducted a systematic and unbiased exploration to identify the optimal modeling strategy. The entire cohort (n=781) was first randomly partitioned into a training set (70% of patients) and an independent test set (30% of patients). Using the Mime R package, we then constructed 101 distinct prognostic models based on the 11 core prognostic genes. These models were generated by creating unique combinations of 10 machine learning algorithms: Lasso, Ridge, Stepwise Cox (stepCox), CoxBoost, Random Survival Forest (RSF), Elastic Net (Enet), Partial Least Squares Regression for Cox (plsRcox), Supervised Principal Components (SuperPC), Generalized Boosted Models (GBM), and Survival Support Vector Machine (survival-SVM).

Each of the 101 combinations represented a distinct pipeline for feature selection and/or model training. All models were developed and tuned exclusively on the training set. For algorithms requiring hyperparameter tuning (e.g., RSF), a 10-fold cross validation was performed only within the training set to identify the optimal parameters. The final selection of the best-performing model was based purely on its predictive accuracy on the unseen independent test set, as measured by the Concordance Index (C-index). The C-index is a key metric for evaluating survival models, quantifying the concordance between the predicted risk order of patients and their actual survival time order. It is the accepted standard for clinical prognostic studies as it effectively handles the censored data common in survival analysis. This data-driven approach ensures that the chosen model has the best-demonstrated generalizability, free from researcher bias.

Patients in the training and test sets were categorized into high-risk and low-risk groups based on their risk scores. The “subplot” function of the “Mime1” package (17) was used to analyze the differences in overall survival (OS) between risk groups (log-rank test, p < 0.05). The diagnostic potential of the histone acetylation-related DEGs was assessed using the “timeROC” (18) and “pROC” packages (19) for analyzing receiver operating characteristic (ROC) curves. Risk score distributions were analyzed and visualized using boxplots.

### 2.6 Prognostic analysis of risk models in different subtypes of LIHC

To further explore the prognostic value of the model, the prognostic ability of the model was assessed using OS in different subgroups. All analyses were done in the “survival” package, and the results were visualized using the “ggplot2” package.

### 2.7 Analysis of differentially expressed genes and enrichment pathways of risk models in LIHC

Differentially expressed genes (DEGs) with corrected p-value < 0.05 and |logFC| > 0.5 were screened based on RNA-seq data. Gene Ontology (GO) and Kyoto Encyclopedia of Genes and Genomes (KEGG) pathway enrichment analysis was performed using the “clusterProfiler” package (20). Based on the logFC-ranked DEGs, the gene set of the MSigDB database (v7.5.1) was used for Gene Set Enrichment Analysis (GSEA). The pathways ranked in the top 10 by normalized enrichment score (NES) were visualized, and correlations between the risk model and core genes within these pathways were analyzed. All visualizations were generated using the “enrichplot” and “ggplot2” packages.

### 2.8 Immune landscape analysis of risk models in LIHC

The “ESTIMATE” package (21) was used to calculate the immunity score, stroma score, and tumor purity for all tumor samples (n=781) and to compare the differences between groups. The relative proportion of immune cells was estimated using the “CIBERSORT” package (22). Immune cell activity was assessed by the “xCell” package (23), and immune cell expression levels were assessed. The correlation of prognostic genes with the immune score, stroma score, and tumor purity was visualized using the “corrplot” package. In addition, risk models were analyzed for correlation with immune checkpoint-related genes, chemokines, chemokine receptors, and immune checkpoint blockade therapy (ICB).

### 2.9 Risk modeling for chemotherapy drug sensitivity analysis in LIHC

Drug sensitivity prediction was performed using the “OncoPredict” package (24). The model was trained based on the GDSC2 dataset, and IC50 values were calculated for the samples. The Wilcoxon test was used to compare the drug sensitivity differences between groups.

### 2.10 Mutational analysis of risk models in LIHC

Based on the somatic mutation data from the TCGA dataset, we used the “maftools” package (25) for mutation analysis. We also analyzed the tumor mutation load (TMB) between the two groups. The frequency of mutations and their distribution in specific genes were analyzed by “maftools” package.

### 2.11 Cross-cohort comparative analysis of mutational landscapes

Molecular data from three hepatocellular carcinoma (HCC) cohorts (TCGA-LIHC, MSK 2024, and CLCA 2024) were obtained from the cBioPortal database (https://www.cbioportal.org/). Mutation types (including point mutations, insertions/deletions) and copy number alterations (CNAs, such as amplifications, deep deletions, shallow deletions) of key genes in the risk model (e.g., HLA-B, NEU1, CDK4) were extracted. The “maftools” package in R software was used to generate an Oncoprint plot visualizing the gene alteration profiles of individual patients across cohorts. A stacked bar plot was created using the “ggplot2” package to compare the overall frequencies of major alteration types (amplification, deep deletion, mutation) among the three cohorts. For the NEU1 gene in the MSK-IMPACT cohort, the Kruskal-Wallis test was performed to compare the distribution of mutation counts across different CNA types (amplification, gain, diploid, shallow deletion; *p* < 0.05 was considered statistically significant).

### 2.12 Correlation analysis of risk models with ferroptosis and m6A methylation-related genes in LIHC

Ferroptosis refers to impaired intracellular lipid peroxide metabolism and toxic lipid production, which induces cell death. m6A is a methylation of RNA, i.e., on the 6th nitrogen atom of adenine (A) in RNA, which affects mRNA stability, translational efficiency, alternative splicing, and localization. We analyzed the prognostic risk model to correlate with ferroptosis and m6A-related genes in LIHC. Ferroptosis-related genes were derived from Ze-Xian Liu et al. Systematic analysis of the abnormalities and functions of ferroptosis in cancer (26). m6A-related genes were derived from Juan Xu et al. on the molecular characterization and clinical significance of m6A regulators across 33 cancer types (27). Statistical significance (*p* < 0.05) was estimated using an independent t-test for normally distributed variables.

### 2.13 NEU1-based analysis of cellular communication

Intercellular communication networks are systematically analyzed and resolved at the cellular level using the “CellChat” (28) package. The method is based on a database of ligand-receptor interactions that quantifies and predicts major cellular communication pathways. A probabilistic statistical framework is used to infer cell type-specific ligand-receptor pair communication probabilities and quantify the strength of communication and the number of receptor-ligand pairs between different cell types. Identify key cellular communication patterns in the tumor microenvironment, with special attention to signaling pathways associated with NEU1 and differences between groups with high and low NEU1 expression.

### 2.14 Pseudotime analysis based on NEU1

The “CytoTRACE” (29) algorithm was used to assess the developmental potential of single cells, and the CytoTRACE score was calculated based on the number of genes detected in each cell, with higher scores indicating cells with higher differentiation potential. The distribution of CytoTRACE scores was compared between tumor epithelial cells and normal epithelial cells to assess the effect of cancer on the differentiation status of cells. In cancer epithelial cell populations, the CytoTRACE scores of high and low NEU1 expression groups were compared to explore the correlation between NEU1 expression levels and cell differentiation potential. The endothelial cell differentiation trajectories were constructed by applying the “Monocle3” package (30), and the cell distribution was visualized by the UMAP downscaling method and the master map was calculated to infer the cell differentiation trajectories. Along the inferred differentiation trajectory, the dynamic changes in the expression of NEU1 and its related genes were analyzed. Combining the proposed temporal information with differential expression analysis, the potential regulatory role of NEU1 in endothelial cell differentiation was revealed.

### 2.15 Molecular docking based on NEU1

The NEU1 gene was mapped by the Coremine Medical Ontology Information Retrieval Platform (www.coremine.com/medical/) to screen its top 10 potential regulatory molecules. Download NEU1 structure files from the PDB database (https://www.rcsb.org/) to obtain target protein result files. Obtain the structure files of active compounds from PubChem database (https://pubchem.ncbi.nlm.nih.gov/), CB-Dock2 platform (31–33) to optimize the structure (removing water molecules, ligands), and molecular docking simulation to evaluate the binding role of receptor proteins to small-molecule ligands after hydrogenation and balancing the charge on receptor proteins.

### 2.16 RNA preparation and quantitative real-time PCR

Total RNA was extracted from tissues using TRIZOL reagent (Invitrogen), and the process was performed according to the instructions. RNA was reversed to cDNA using the PrimeScript RT kit (Takara). qRT-PCR experiments were completed using the Takara SYBR Premix Ex Taq II kit, and the process was performed according to the instructions. The final results were corrected for the expression of GAPDH.

The primer sequences used in this study were as follows:

GAPDH-forward: TGTGGGCATCAATGGATTTGG
GAPDH-reverse: ACACCATGTATTCCGGGTCAAT
HLA-B-forward: TCCTAGCAGTTGTGGTCATC
HLA-B-reverse:TCAAGCTGTGAGAGACACAT
HEXB-forward: GATGTTGGCGCTGCTGACTC
HEXB-reverse: GGGCTGTGGCTGATGTAGAA
CDK4-forward: AATGTTGTACGGCTGATGGA
CDK4-reverse: AGAAACTGACGCATTAGATCCT
ACAT1-forward: CTGGGTGCAGGCTTACCTAT
ACAT1-reverse: ACATGCTCTCCATTCCACCTG
NAA10-forward: ATGAACATCCGCAATGCGAG
NAA10-reverse: CTAGGAGGCTGAGTCGGAGG
B2M-forward: TGCTGTCTCCATGTTTGATGTATCT
B2M-reverse: TCTCTGCTCCCCACCTCTAAGT
HSPD1-forward: TTGACTGCCACAACCTGAAG
HSPD1-reverse: CACCGTAAGCCTTTGGTCAT
NPM1-forward: GGAGGTGGTAGCAAGGTTCC
NPM1-reverse: TTCACTGGCGCTTTTTCTTCA
PON1-forward: CTGCTGATTGGCACAGTGTT
PON1-reverse: GGGTCAGCATTCATTGTTCA
NEU1-forward: TGAAGTGTTTGCCCCTGGAC
NEU1-reverse: AGGCACCATGATCATCGCTG
CFB-forward: GGAAGGGAATGTGACCAGG
CFB-reverse: AAGGCAGGAGAGAAGCTGG

### 2.17 Western blotting

Frozen tissues were lysed using RIPA buffer (Beyotime, China) containing a protease inhibitor mixture. Protein concentration was detected using the BCA Protein Quantification Kit (Beyotime, China). Protein samples were separated on 8% SDS-PAGE gels and transferred to PVDF membranes blocked with 5% non-fat powdered milk at low temperature for 2 h. The PVDF membranes were incubated with diluted primary antibodies and incubated overnight at 4°C (NEU1: 1:2000, proteintech, USA; GAPDH: 1:1000, CST, USA).

On the following day, after three washes with TBST, the PVDF membranes were incubated with HRP-conjugated secondary antibody (1:2000) for 2 hours. After three washes with TBST, protein bands were visualized using an ECL detection kit (Thermo Fisher, USA; 32106) and imaged with a Bio-Rad ChemiDoc Imaging System. Band intensity was quantified using ImageJ software.

### 2.18 NEU1 knockdown and validation

NEU1-specific siRNA (Santa Cruz Biotechnology, sc-106297) was transfected into Hep3B and MHCC-97H cell lines using Lipofectamine™ 3000 (Thermo Fisher Scientific, L3000008) to knock down NEU1 expression. Knockdown efficiency was assessed by RT-qPCR and Western blotting.

For RT-qPCR analysis, cells were seeded in 12-well plates and transfected with 40 pmol siRNA per well. Total RNA was isolated 24 h post-transfection using TRIzol reagent (Invitrogen) and reverse-transcribed into cDNA with a PrimeScript RT kit (TaKaRa, RR036A). qPCR amplification was performed in 10 μL reactions using SYBR Green PCR Master Mix (TaKaRa, RR420B). Melting curve analysis confirmed primer specificity. The relative mRNA expression levels were normalized to GAPDH and calculated using the 2−ΔΔCt method. All primer sequences were synthesized by Sangon Biotech (Shanghai, China).

For Western blotting, cells were harvested 48 h after transfection. Total protein was extracted using RIPA lysis buffer (Beyotime, China; P0013B) supplemented with a protease inhibitor cocktail (Roche, Switzerland; 4693116001; 1 mg/mL). Protein concentrations were quantified using a BCA assay kit (Beyotime; P0010), and 20-30 μg of protein per sample was separated on 8% SDS-PAGE gels. Proteins were transferred to PVDF membranes (Millipore, USA), followed by blocking with 5% non-fat powdered milk for 2 h at room temperature. Membranes were incubated overnight at 4°C with primary antibodies against NEU1 (Proteintech, 67032-1-Ig; 1:1000) and GAPDH (Proteintech; 60004-1-Ig; 1:3000), followed by incubation with HRP-conjugated goat anti-rabbit secondary antibodies for 2 h at room temperature. Protein bands were visualized using an ECL detection kit (Thermo Fisher, USA; 32106) and imaged with a Bio-Rad ChemiDoc Imaging System. Band intensity was quantified using ImageJ software.

### 2.19 CCK-8 assay

Transfected Hep3B and MHCC-97H cells were plated in 96-well culture plates at a density of 2×10³ cells per well. Following incubation under standard culture conditions (37°C, 5% CO_2_) for 12, 24, and 36 hours, the culture medium was replaced with 100 μL of DMEM containing CCK-8 reagent (Beyotime Biotechnology, China; C0038) at a 1:10 (v/v) dilution. After 1 hour of additional incubation, optical density measurements were performed using a microplate reader at a wavelength of 450 nm.

### 2.20 Colony formation assay

Transfected Hep3B and MHCC-97H cells (1000–2000 cells/well) were plated in 6-well plates and cultured in a complete medium for 14 days. Colonies were fixed with 4% paraformaldehyde for 20 min, stained with 1% (w/v) crystal violet (Beyotime, China; C0121) for 20 min, and washed three times with deionized water. Colonies containing more than 50 cells were counted using an inverted microscope.

### 2.21 Wound healing assays

The ibidi Culture-Insert 2 Well (ibidi GmbH, Germany, 80209) was placed in a 60-mm Petri dish. Transfected Hep3B and MHCC-97H cells were seeded into the left and right chambers of the insert at a density of 2.5×10^5^ cells/well, respectively. After 12 hours of incubation in a serum-free medium, the culture insert was carefully removed. Cell migration patterns were documented at 0 h and 24 h using an inverted microscope.

### 2.22 Cell invasion assays

Transwell inserts (Corning, USA; 3422) were coated with Matrigel matrix (Beyotime, China; Cat# C0376-5ml) diluted in DMEM at a 1:8 ratio (v/v). After 12 hours of serum starvation, cells were trypsinized and resuspended in a serum-free medium. Subsequently, 5×10^4^ cells per well were seeded in the upper chamber, while the lower chamber was filled with 600 μL of complete medium. Following 36 hours of incubation, cells were fixed with 4% paraformaldehyde for 20 minutes and stained with 1% crystal violet (Beyotime, China; C0121) for 20 minutes. After being washed three times with deionized water, non-invading cells on the upper membrane surface were gently removed using cotton swabs. Migrated cells were imaged with an inverted microscope, and five random fields per well were analyzed.

### 2.23 Statistical analysis

All statistical analyses and visualizations were done by RStudio (version 4.3.3) and GraphPad Prism 8.0. Comparisons of normally distributed continuous variables between two groups were performed using the independent samples t-test, and non-normally distributed variables were tested using the Wilcoxon rank sum test. The Welch one-way ANOVA was used to evaluate comparisons between several groups. Each experiment was repeated three times, and all *in vivo* experiments were performed with at least five animals per group. Data are expressed as mean ± standard error of the mean (SEM), with *p* < 0.05 considered statistically significant.

## 3 Result

### 3.1 Acquisition and processing of single-cell datasets

**Figure 1:**
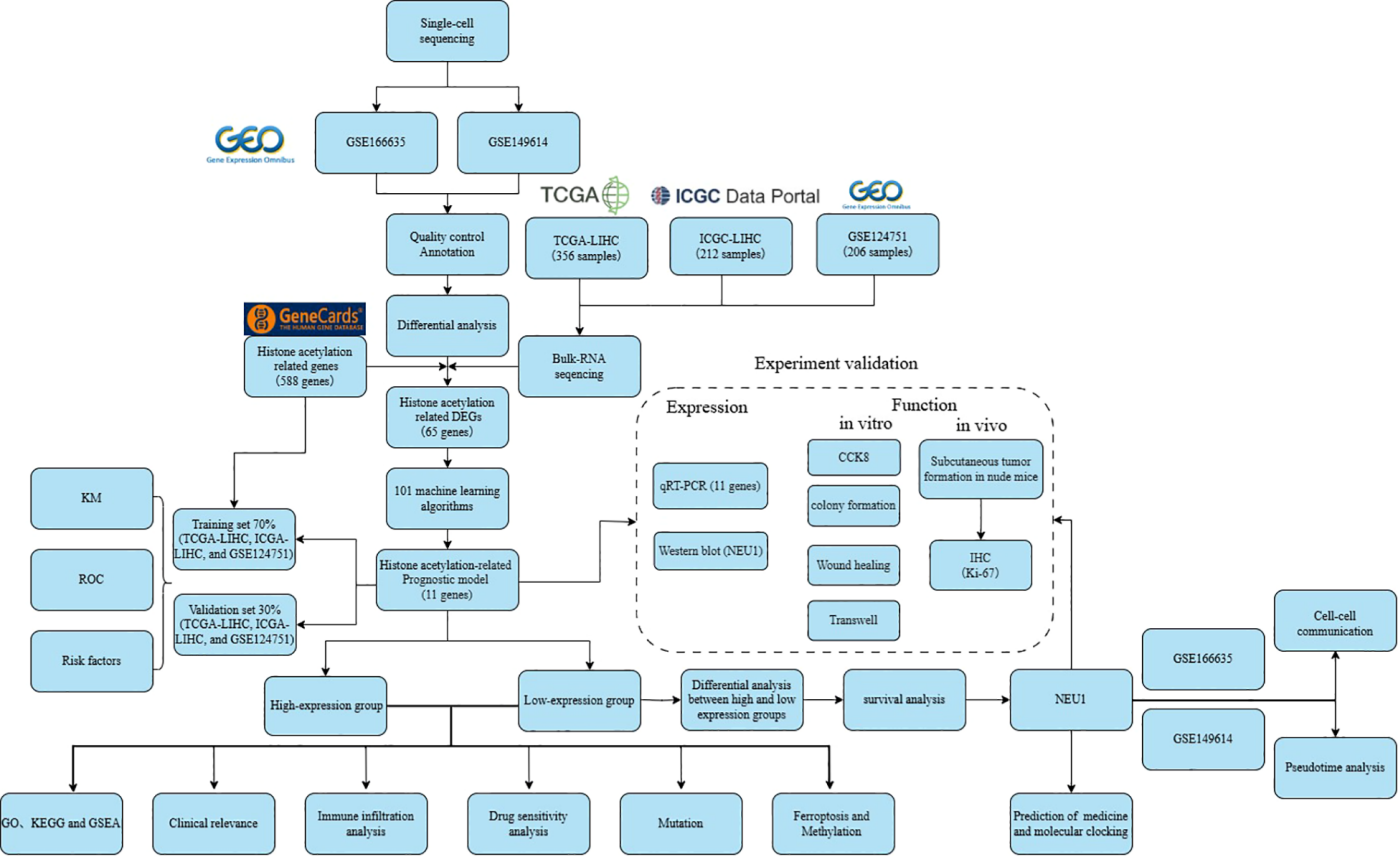
Systematic workflow.

**Figure 2:**
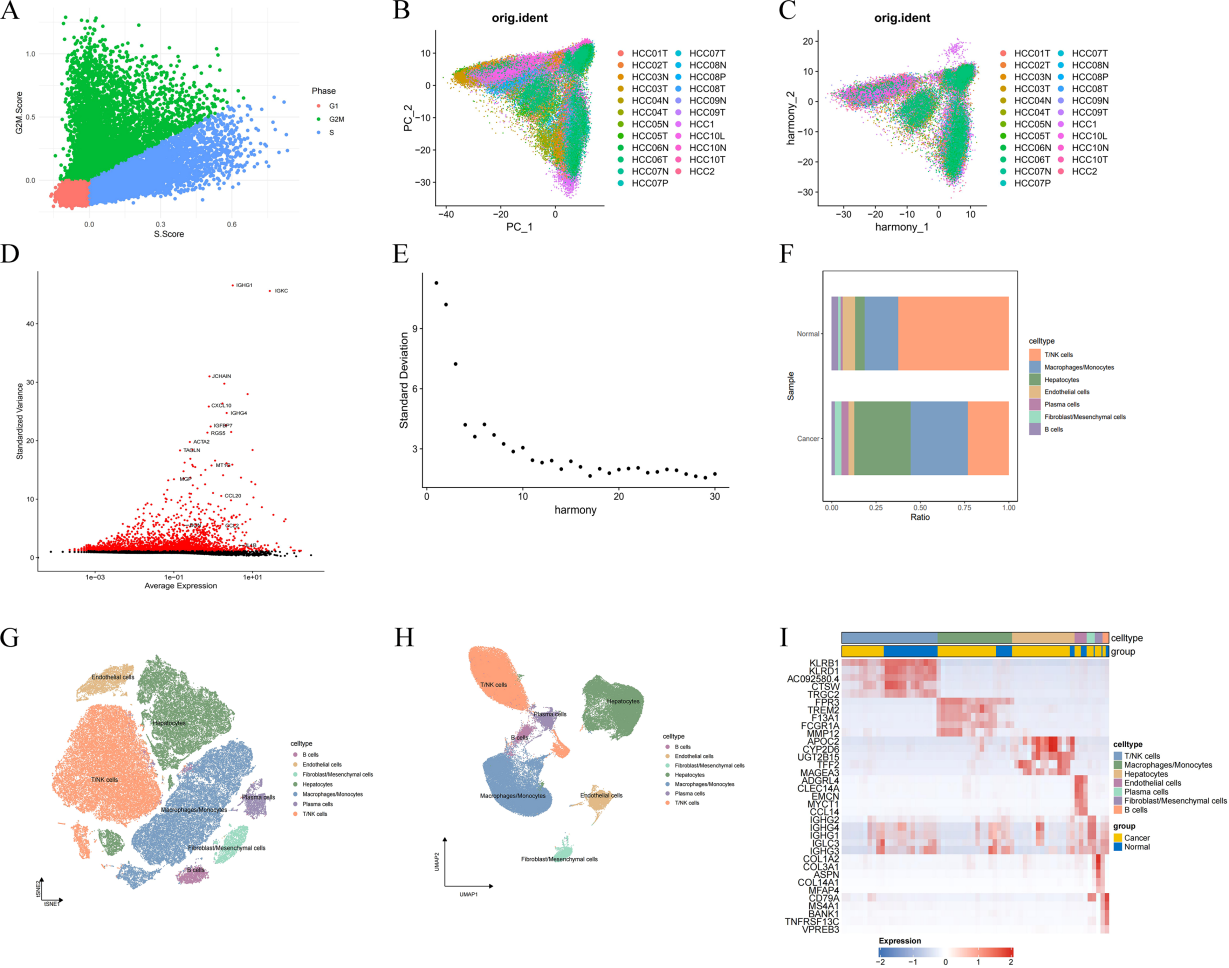
Acquisition of LIHC single-cell datasets. **(A)** Cell cycle visualization; **(B)** Harmony batch-corrected pre-decimation plots; **(C)** Harmony batch-corrected post-decimation plots; **(D)** the top 15 highly variable signature genes; **(E)** Harmony post-decimation principal component ANOVA contribution analysis plots; **(F)** the cellular compositions in the normal tissues and hepatocellular carcinoma tissues; **(G)** the cellularly annotated t-SNE plots; **(H)** the cellularly annotated UMAP plot; **(I)** Heatmap of the first 5 genes of each cell cluster in normal and hepatocellular carcinoma tissue.

**Figure 1** summarizes the overall workflow of this study. We first performed quality control and downstream analysis on two datasets, GSE166635 and GSE149614, from the Gene Expression Omnibus (GEO) database, and filtered the data to obtain a total of 86,409 cells, which were categorized into three groups of G1, G2M, and S phases, respectively (**Figure 2A**). Subsequently, the “RunHarmony” function was applied for batch removal, and principal component analysis (PCA) was used for dimensionality reduction, which successfully removed the batch effect while preserving the differences in the data (**Figures 2B, C**). We further analyzed the highly variable genes in the samples, and the results showed that 15 genes, including IGHG1 and IGKC, were significantly enriched in the high-expression-high-variation region (**Figure 2D**). Using the “ElbowPlot” function, we found that the standard deviation curve flattened out after the harmony dimension was about 10, indicating that the contribution of additional dimensions to the variation of the data gradually decreased. Therefore, we chose the first 10 harmony dimensions for downstream clustering and dimensionality reduction visualization analysis, balancing the trade-off between dimensionality reduction and computational complexity (**Figure 2E**). Following unsupervised clustering of the scRNA-seq data, we employed UMAP dimensionality reduction for visualization. This process partitioned the cells into seven distinct clusters, which were subsequently annotated based on canonical marker genes as: B cells, Endothelial cells, Fibroblast/Mesenchymal cells, Hepatocytes, Macrophages/Monocytes, Plasma cells, and T/NK cells. To demonstrate the effectiveness of our cross-platform normalization for the bulk transcriptomic data, we performed UMAP on the integrated patient samples. The resulting plot shows a homogenous mixture of samples from all cohorts, confirming that batch effects were successfully removed. Finally, analysis of cellular composition revealed that Hepatocytes, Macrophages/Monocytes, and T/NK cells were the most abundant populations in LIHC samples, whereas T/NK cells and Macrophages/Monocytes were the dominant populations in normal liver tissue (**Figures 2F-H**, **Supplementary Figure 1**).

In addition, to leverage the high resolution of single-cell RNA sequencing, we performed a cell-type-specific differential expression analysis. Specifically, within each annotated cell type (e.g., T cells, hepatocytes), we compared cells originating from LIHC tumor tissue with those from adjacent normal tissue. The lists of differentially expressed genes from each cell-type comparison were then merged and deduplicated, yielding a final set of 1,625 unique DEGs for subsequent analysis (**Figure 2I**, **Supplementary Table 1**).

### 3.2 Constructing and validating histone acetylation-related risk models with 101 machine learning combinations in LIHC

**Figure 3:**
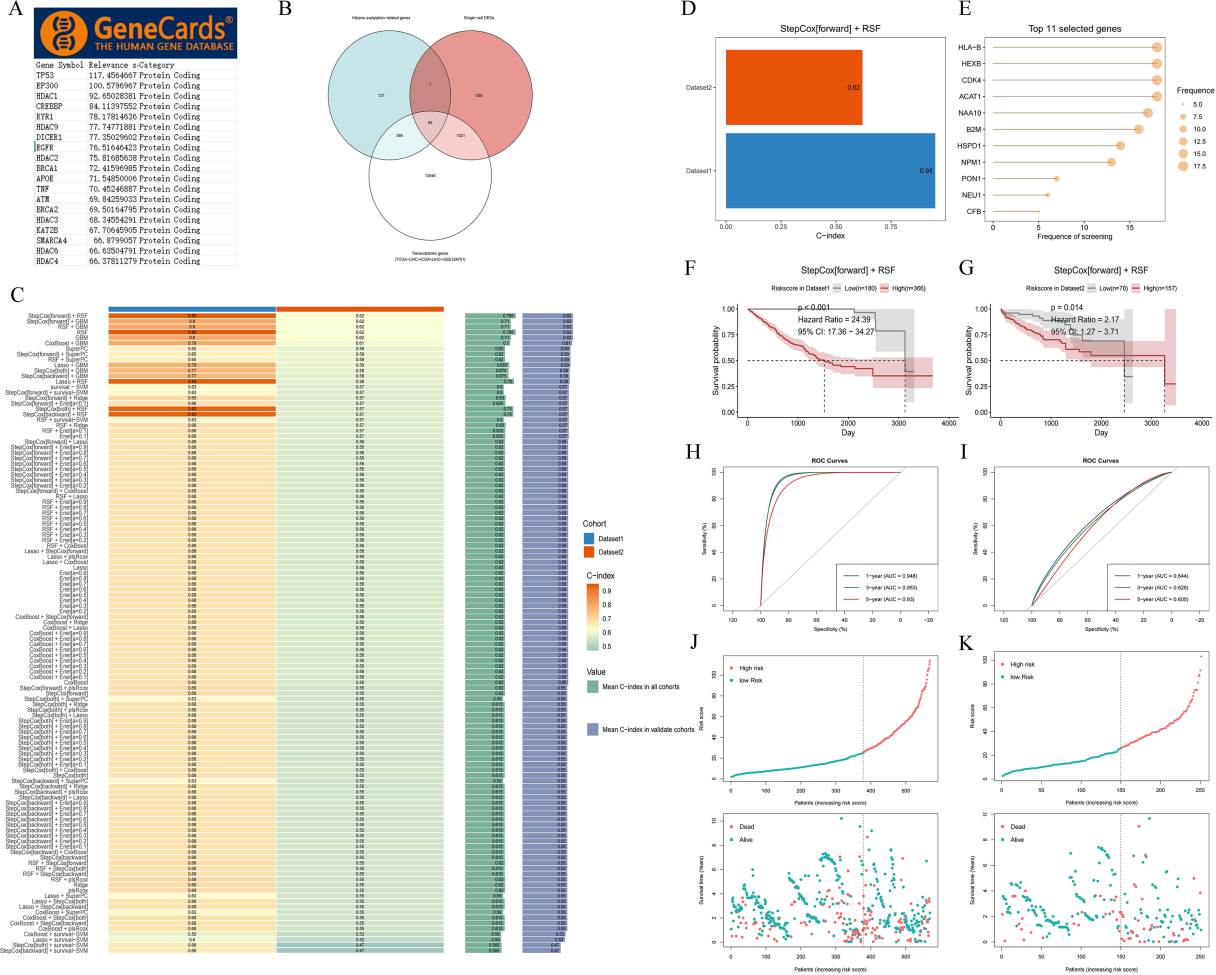
Construction and validation of a histone acetylation-associated risk model for LIHC by 101 machine learning combinations. **(A)** Acquisition of histone acetylation gene sets from GeneCards; **(B)** Acquisition of 65 Histone acetylation-related DEGs; **(C)** 101 combinations of machine learning algorithms and their average C-indexes; **(D)** C-indexes of models constructed by the combination of StepCox[forward] + RSF in the training and validation sets; **(E)** C index; **(E)** 11 risk genes (HLA-B, HEXB, CDK4, ACAT1, NAA10, B2M, HSPD1, NPM1, PON1, NEU1, and CFB) included in the models constructed by the StepCox[forward] +RSF combination; **(F, G)** prognostic value of the risk models in the training and validation sets; **(H, I)** Time-dependent ROC of the risk model in the training and validation sets; **(J, K)** Risk factor maps of the risk model in the training and validation sets.

To identify a set of candidate prognostic genes for subsequent analysis, we implemented a multi-step filtering process. We began with a list of 588 genes highly associated with histone acetylation, obtained from the GeneCards database (**Figure 3A**, **Supplementary Table 2**). Next, to ensure these genes were not only biologically relevant and actively dysregulated in the liver cancer microenvironment, but also measurably present in our study cohorts, we performed a crucial intersection. We intersected these 588 genes with both (1) a list of 1,625 differentially expressed genes (DEGs) identified from single-cell analysis, and (2) the set of all genes available in our combined transcriptomic dataset (TCGA, ICGC, and GSE). This rigorous process yielded a final set of 65 genes, which were defined as histone acetylation-related differentially expressed genes (Hac-DEGs) and were carried forward for all subsequent prognostic model development (**Figure 3B**, **Supplementary Table 3**).

Next, we went through 101 machine-learning combinations and obtained the C-index of each combination in all training and validation sets. The results showed that the StepCox[forward]+RSF combination had the highest C-index, which was 0.95 and 0.62 in the training and validation sets, respectively, suggesting that it had the best performance in predicting the prognosis of LIHC (**Figures 3C, D**). As a result, a histone acetylation-associated risk model containing 11 genes (HLA-B, HEXB, CDK4, ACAT1, NAA10, B2M, HSPD1, NPM1, PON1, NEU1, and CFB) was constructed (**Figure 3E**). We evaluated the expression of these 11 genes in the TCGA-LIHC dataset (**Supplementary Figure 2**) and their respective roles in assessing the overall survival (OS), disease-specific survival (DSS), progression-free interval (Progression Free Interval (PFI), and Disease-free-survival (DFS) (**Supplementary Figure 3**).

A risk score was assigned to each patient using the model, and all patients were included in either the high-risk or low-risk group based on the score. The OS of patients in different subgroups was analyzed in the training set using KM curves, and the results showed that patients in the high-risk group had a significantly worse prognosis (p<0.001, 95% CI:17.36-34.27); this result was also confirmed in the validation set (p=0.014, 95% CI:1.27-3.71), which suggests that the model demonstrates robust performance in evaluating the overall survival of LIHC patients. (**Figures 3F, G**). In addition, time-dependent ROC analysis showed that the model also had high value in predicting the overall survival of LIHC patients at 1, 3, and 5 years in both the training and validation sets (AUC: 0.948, 0.953, and 0.930 for the training set; AUC: 0.644, 0.628, and 0.605 for the validation set) (**Figures 3H, I**). We depicted the risk factor plots of the model in the training and validation sets, respectively, to demonstrate the relationship between the model score and the survival status of the LIHC patients, where green dots indicate the survival status and red dots indicate the death status. In both the training and validation sets, the number of red points increased as the risk score increased, indicating that patients with high scores on the model had a higher risk of death (**Figures 3J, K**).

Moreover, we incorporated an additional independent validation cohort, OEP000321, to verify the generalization ability of the models. In this rigorous external validation, the StepCox[forward] + RSF model achieved an AUC of 0.74, demonstrating the highest predictive accuracy among candidate models (**Supplementary Figures 4A, B**).

Instead of relying on a single metric, we evaluated the stability of the models over time. We calculated the average time-dependent AUC across all three datasets (training set, testing set, and the new OEP000321 validation set) for the 101 machine learning combinations. At the 1-year horizon, the StepCox[forward] + RSF model ranked 2nd among all combinations (**Supplementary Figure 4C**). At the 3-year horizon, the model ranked 1st, demonstrating superior mid-term predictive power (**Supplementary Figure 4D**). At the 5-year horizon, the model also ranked 1st, further confirming its long-term robustness (**Supplementary Figure 4E**).

### 3.3 Prognostic analysis of risk models in different subtypes of LIHC

**Figure 4:**
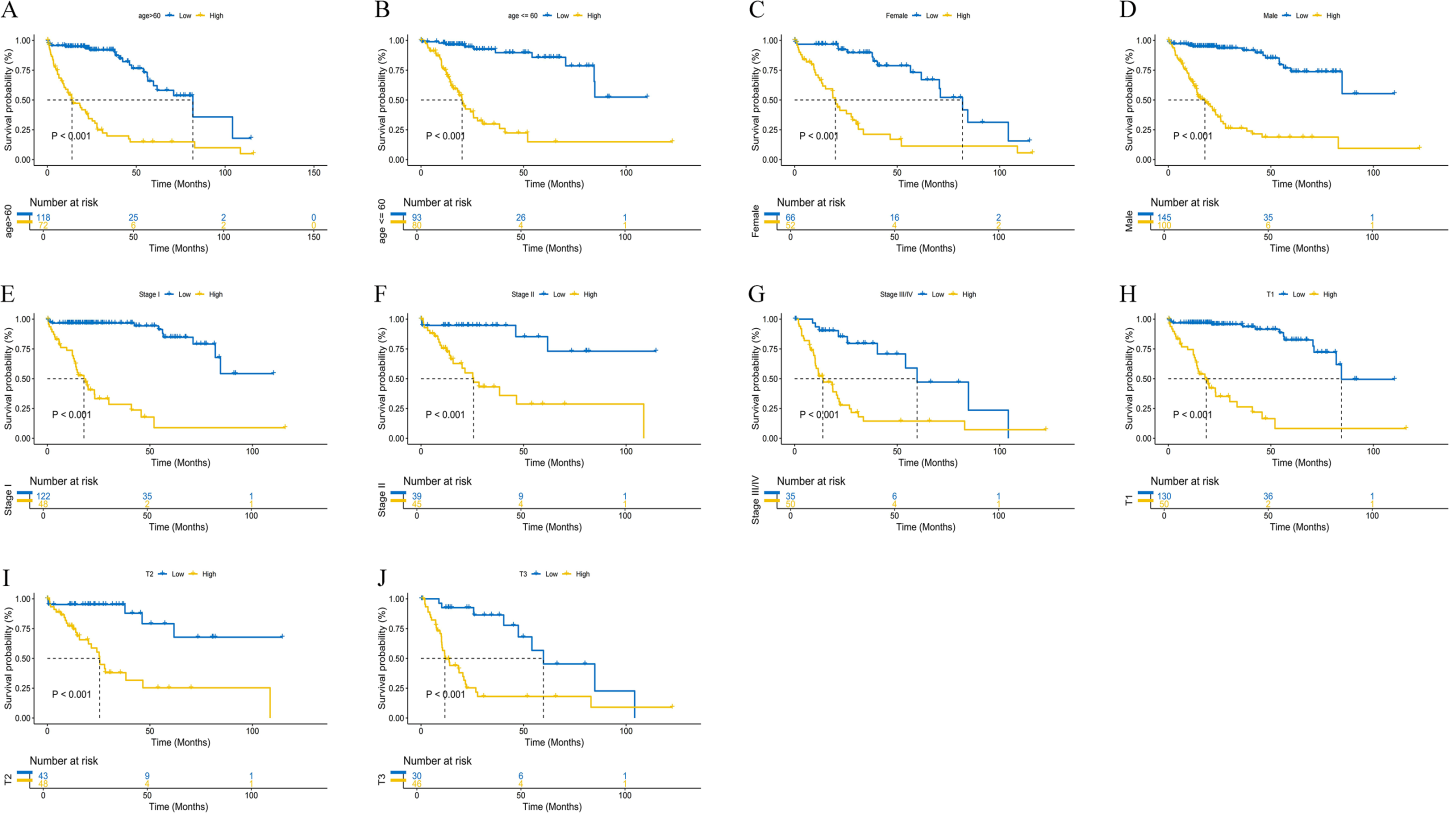
Prognostic value of the histone acetylation-related risk model in different subgroups of LIHCPrognostic value of histone acetylation-associated risk model in **(A)** age >60; **(B)** age ≤60; **(C)** female; **(D)** male; **(E)** Stage I; **(F)** Stage II; **(G)** Stage III/IV; **(H)** T1; **(I)** T2; **(J)** T3.

We evaluated the prognostic value of the risk model in different subgroups of TCGA-LIHC using R software. The results showed that the model had strong prognostic significance across ten distinct subgroups stratified by age (>60 or ≤60 years), sex (female or male), pathologic stage (I, II, or III/IV), and T stage (T1, T2, or T3) (all *p* < 0.001; **Figure 4**). We then performed survival analysis across clinical subgroups within the entire ICGC-LIRI-JP cohort (**Supplementary Figure 5**) and validated the prognostic performance in the clinical subgroups of the entire GSE124751 cohort (**Supplementary Figure 6**). These findings suggest that this model has strong prognostic value in LIHC and is expected to be another new model for predicting the prognosis of LIHC patients.

### 3.4 Analysis of differentially expressed genes and enrichment pathways of risk models in LIHC

**Figure 5:**
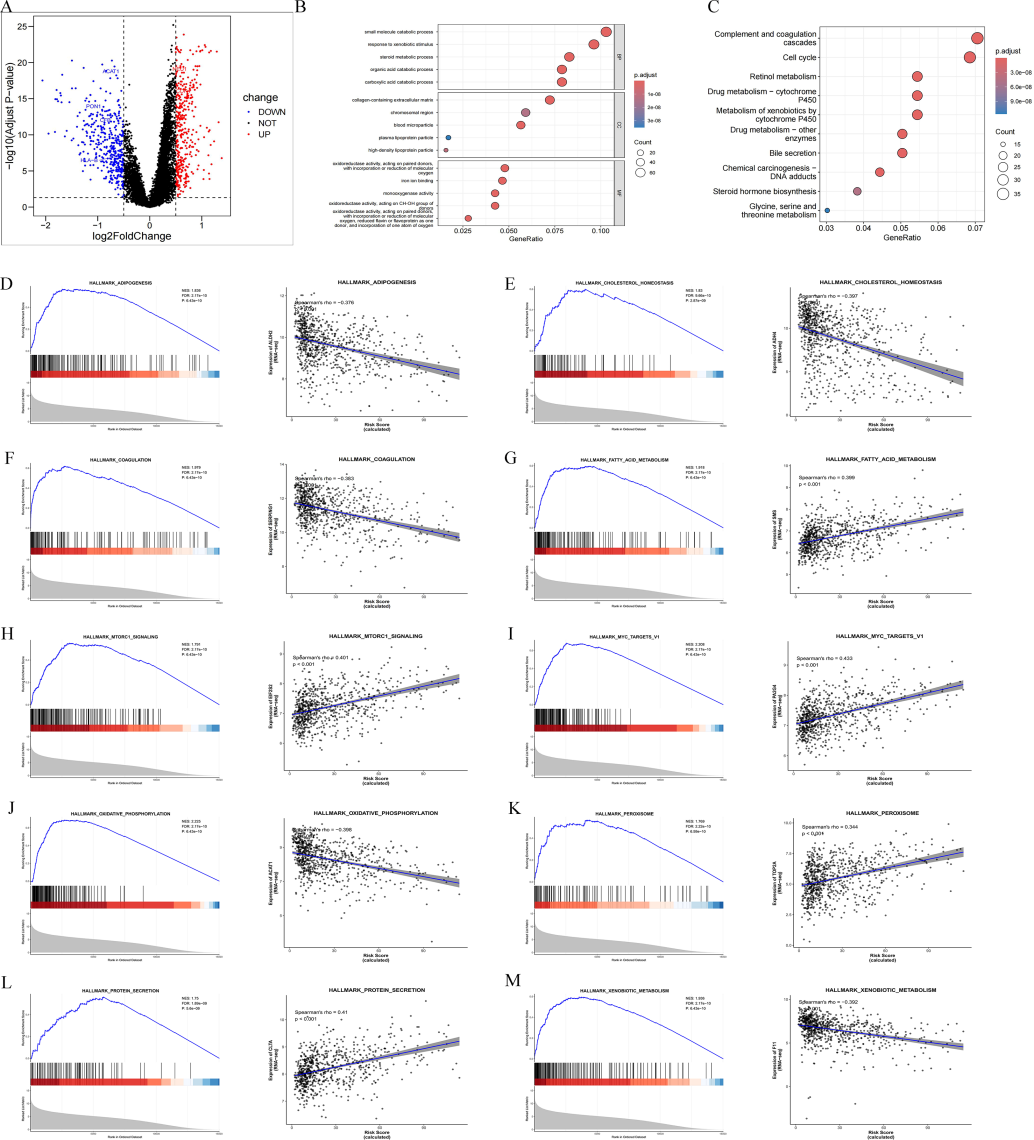
Differential gene and enrichment pathway analysis of histone acetylation-related risk model in LIHC. **(A)** Differential gene volcano maps of histone acetylation-associated risk models; **(B)** GO analysis of differential genes; **(C)** KEGG analysis of heterozygotes; **(D–M)** GSEA analysis of differential genes and correlation analysis of histone acetylation-associated risk model scores with core genes of each pathway.

We divided 781 LIHC patients into high-risk and low-risk groups based on the scoring of the risk model, compared the differential genes between the two groups in R software, and screened a total of 768 differential genes, including 367 up-regulated genes and 401 down-regulated genes (**Figure 5A**). To understand the potential enrichment pathways of these genes, we performed GO and KEGG enrichment analyses. The results of GO analysis showed that these differential genes were mainly enriched in the small molecule catabolic process, response to xenobiotic stimulus, and collagen-containing extracellular matrix (**Figure 5B**). KEGG analysis showed that these differential genes were mainly enriched in Complement and coagulation cascades, Cell cycle, and Retinol metabolism pathways (**Figure 5C**). GSEA enrichment analysis showed that the model was significantly associated with some pathways in LIHC, e.g., ADIPOGENESIS (NES = 1.836, *p* < 0.001), CHOLESTEROL HOMEOSTASIS (NES = 1.83, *p* < 0.001), COAGULATION (NES = 1.979, *p* < 0.001), FATTY ACID METABOLISM (NES = 1.918, *p* < 0.001), MTORC1_SIGNALING (NES = 1.791, *p* < 0.001), MYC_TARGETS_V1 (NES = 2.208, *p* < 0.001), OXIDATIVE PHOSPHORYLATION (NES = 2.225, *p* < 0.001), PEROXISOME (NES = 1.769, *p* < 0.001), PROTEIN SECRETION (NES = 1.75, *p* < 0.001), XENOBIOTIC METABOLISM (NES = 1.936, *p* < 0.001), and the model scores were significantly correlated with the core genes of the above pathways (ALDH2: rho = -0.376, *p* < 0.001; ADH4: rho = -0.397, *p* < 0.001; SERPING1: rho = -0.383, *p* < 0.001; SMS: rho=0.399, *p* < 0.001; EIF2S2: rho=0.401, *p* < 0.001; PA2G4: rho=0.433, *p* < 0.001; ACAT1: rho=-0.398, *p* < 0.001; TOP2A: rho=0.344, *p* < 0.001; CLTA: rho=0.41, *p* < 0.001; F11: rho=-0.392, *p* < 0.001) (**Figures 5D–M**). These results suggest that the modulation of downstream pathways by this model may be by targeting these molecules to produce positive or negative effects, providing new targets for subsequent anti-LIHC drug development.

### 3.5 Immune landscape analysis of risk models in LIHC

**Figure 6:**
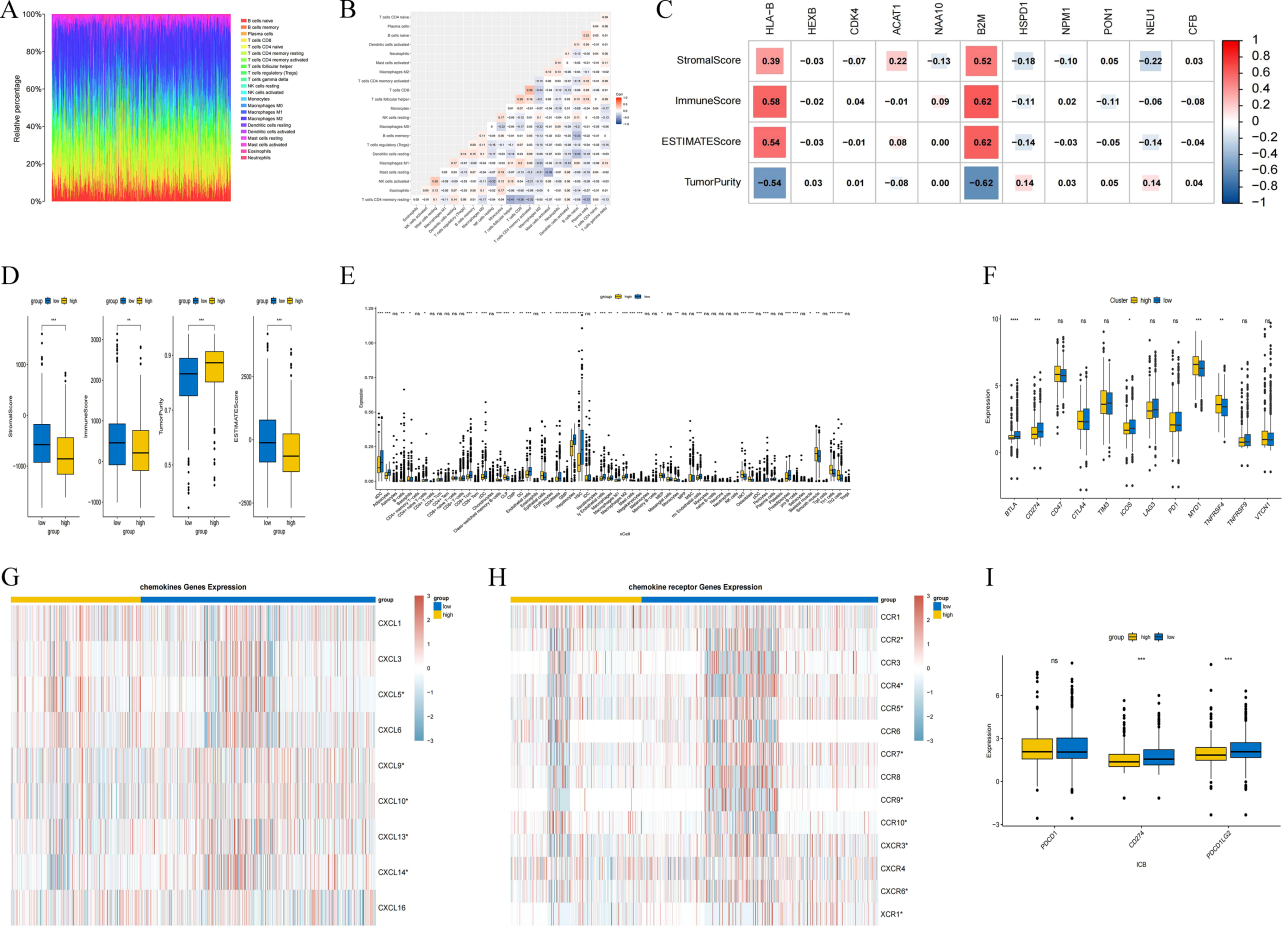
Analysis of immune infiltration in LIHC by the histone acetylation-related risk model. **(A)** Compositional analysis of immune cells in all samples; **(B)** Correlation analysis between immune cells in all samples; **(C)** Correlation analysis between the expression of each of the 11 risk genes and stromal score, immune score, ESTIMATEScore, and tumor purity; **(D)** Scoring of the histone acetylation-associated risk model with a stromal score, immune score, ESTIMATEScore, and tumor purity; **(E)** correlation analysis with immune cell infiltration; **(F)** correlation analysis with immune checkpoints; **(G)** correlation analysis with chemokines; **(H)** correlation analysis with chemokine receptors; and **(I)** assessment of immunotherapeutic response based on the TIDE algorithm.(*p < 0.05; **p < 0.01; ***p < 0.001; ****p < 0.0001).

We first analyzed the composition of immune cells in all samples using the CIBERSORT algorithm with R software. The results showed that the immune cells in the samples were predominantly T cells and macrophages, with the highest percentage of M0-type macrophages and the smallest percentage of neutrophils (**Figure 6A**). Analysis of the correlation between immune cells in the microenvironment revealed a significant positive correlation between CD4 memory activated T cells and CD8 T cells (Cor=0.36), as well as a significant negative correlation between CD4 follicular helper T cells and CD4 memory resting T cells (Cor=-0.41) (**Figure 6B**). Examination of the correlation between each gene in the model and tumor stroma score, immune score, ESTIMATEScore, and tumor purity demonstrated a strong correlation between HLA-B and B2M with the tumor stroma score, immune score, ESTIMATEScore, and tumor purity (**Figure 6C**). Further analysis indicated that a higher model score corresponded to greater tumor purity and lower tumor stroma score, immune score, and ESTIMATEScore (*p* < 0.01) (**Figure 6D**).

We analyzed the correlation between the risk model score and immune cell infiltration, immune checkpoint-related gene expression, chemokine and chemokine receptor infiltration with R software, and the results showed that the infiltration level of many kinds of immune cells in the microenvironment changed significantly with the higher risk score of the model, the expression level of immune checkpoint-related genes, such as MYD1, TNFRSF4, and other immune checkpoint-related genes, were significantly up-regulated. The infiltration levels of chemokines such as CXCL5, CXCL9, CXCL10, CXCL13, CXCL14, and chemokine receptors such as CCR2, CCR4, CCR5, CCR7, CCR9, CCR10, CXCR3, CXCR6, XCR1, etc., also changed significantly (*p* < 0.05) (**Figures 6E–H**).

To assess the clinical potential of risk models in immunotherapy, we analyzed the ICI responses of the high-risk and low-risk groups using the Tumor Immune Dysfunction and Exclusion (TIDE) algorithm with R software. The results showed that the ICB scores for CD274 and PDCD1LG2 were lower in the high-risk group, indicating that the high-risk group had a better response to targeted CD274 and PDCD1LG2 therapy (*p* < 0.001) (**Figure 6I**).

### 3.6 Risk modeling for chemotherapy drug sensitivity analysis in LIHC

**Figure 7:**
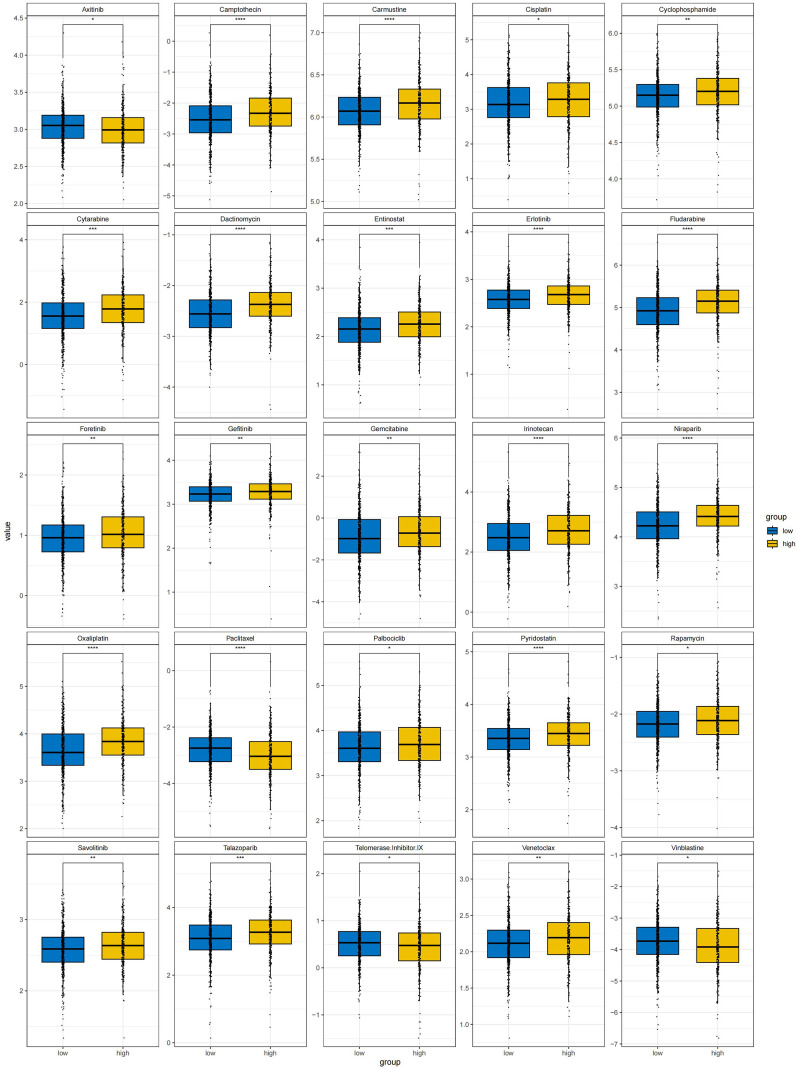
Histone acetylation-related risk model for correlation with chemotherapeutic drug sensitivity in LIHC (*p < 0.05; **p < 0.01; ***p < 0.001; ****p < 0.0001).

We analyzed the correlation between risk model scores and sensitivity to 25 clinically used chemotherapeutic drugs based on the GDSC2 database. The results demonstrated that the higher the risk model score, the lower the IC50 of Axitinib, Paclitaxel, Telomerase Inhibitor IX, and Vinblastine, indicating greater sensitivity to these drugs. Conversely, for Camptothecin, Carmustine, Cisplatin, Cyclophosphamide, Cytarabine, Dactinomycin, Entinostat, Erlotinib, Fludarabine, Foretinib, Gefitinib, Gemcitabine, Irinotecan, Niraparib, Oxaliplatin, Palbociclib, Pyridostatin, Rapamycin, Savolitinib, Talazoparib, and Venetoclax, higher IC50 values correlated with an increased susceptibility to develop resistance to these drugs (*p* < 0.05) (**Figure 7**). These findings suggest that risk model scores are instructive for personalized therapeutic drug selection in patients with LIHC.

### 3.7 Mutational analysis of risk models in LIHC

**Figure 8:**
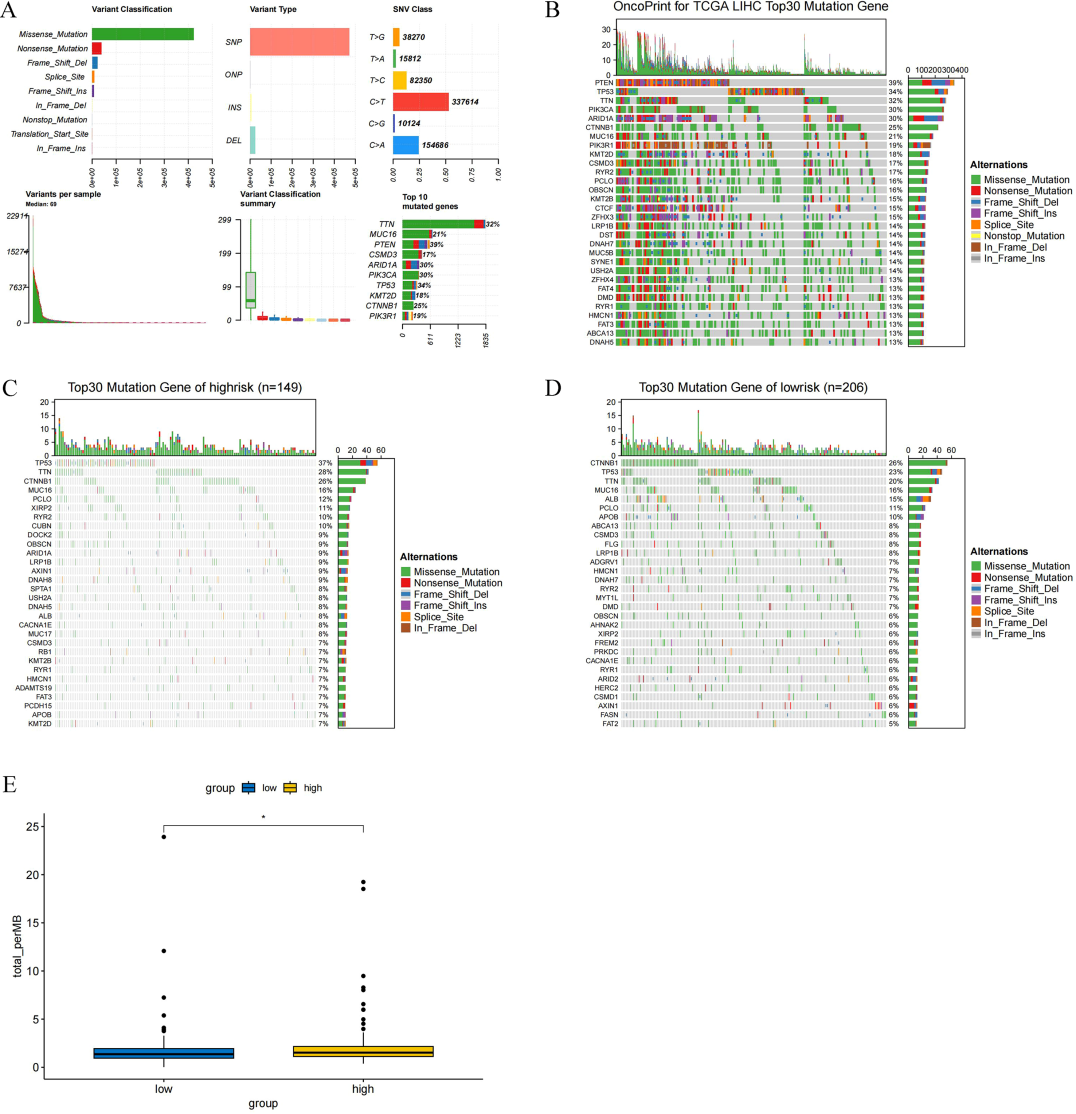
Mutation analysis of histone acetylation-related risk models in LIHC **(A)** Analysis of mutation types; **(B)** Top 30 genes with the highest mutation frequency in all samples; **(C)** Top 30 genes with the highest mutation frequency in samples from the high-risk group; **(D)** Top 30 genes with the highest mutation frequency in samples from the low-risk group; **(E)** TMB analysis between the high-risk and low-risk groups. (*p < 0.05).

Based on the somatic mutation data from the TCGA-LIHC dataset, we analyzed the mutational profiles associated with the risk model using R software. The results showed that missense mutation was the most common type of alteration (**Figure 8A**). The top 30 most frequently mutated genes in the entire cohort, the low-risk group (n = 149), and the high-risk group (n = 206) are presented in **Figures 8B–D**, respectively.

Tumor mutation burden (TMB) was defined as the total number of somatic gene coding errors, base substitutions, gene insertion or deletion errors detected per million bases. We analyzed the TMB scores between the high-risk and low-risk groups of the risk model and showed that the high-risk group had higher TMB scores (*p* < 0.05) (**Figure 8E**).

To systematically investigate the mutation frequency and spectrum of key genes in our model across diverse molecular contexts, we performed a direct comparison using cBioPortal between the model’s development cohort (TCGA-LIHC) and two other large-scale HCC studies (MSK 2024 and CLCA 2024). This comparative analysis revealed distinct molecular architectures among the three cohorts. The TCGA cohort was characterized by a high frequency of Copy Number Alterations (CNAs), predominantly gene amplifications. In contrast, the MSK 2024 cohort exhibited a mutation-driven profile, featuring specific alterations such as truncating mutations in HLA-B. The CLCA 2024 cohort displayed a relatively genomically inactive background with the lowest overall frequency of CNAs (**Supplementary Figure 7**).

### 3.8 Correlation analysis of risk models with ferroptosis and m6A methylation-related genes in LIHC

**Figure 9:**
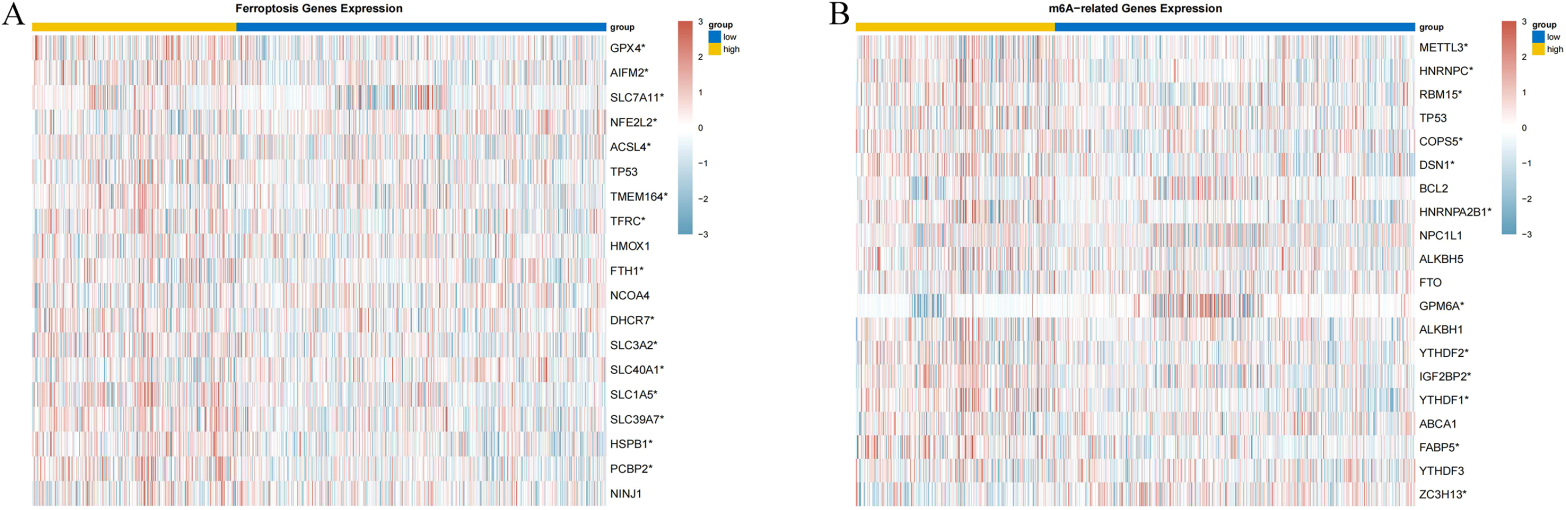
Correlation analysis of histone acetylation-associated risk model with iron death and m6A methylation in LIHC **(A)** Heatmap of correlation between risk model score and iron death-related genes; **(B)** Heatmap of correlation between risk model score and m6A methylation-related genes.

We analyzed the correlation between the risk model score and ferroptosis-related genes, and the results showed that the higher the risk model score, the higher the expression levels of GPX4, AIFM2, SLC7A11, GSS, ACSL4, TMEM164, TFRC, FTH1, DHCR7, SLC3A2, SLC1A5, SLC39A7, HSPB1, PCBP2 were upregulated, while the expression levels of NFE2L2 and SLC40A1 were downregulated (*p* < 0 05), suggesting that the risk model may promote or inhibit ferroptosis by influencing the expression of these genes (*p* < 0 05). were also correspondingly up-regulated, while the expression levels of NFE2L2 and SLC40A1 were correspondingly down-regulated (*p* < 0.05), suggesting that the risk model may promote or inhibit ferroptosis by affecting the expression of these genes (**Figure 9A**).

We performed a correlation analysis between risk model scores and m6A methylation-related genes, and the results showed that the higher the risk model scores, the expression levels of METTL3, HNRNPC, RBM15, COPS5, DSN1, HNRNPA2B1, YTHDF2, IGF2BP2 and FABP5 were correspondingly up-regulated, whereas the expression levels of GMP6A, and ZC3H13 were correspondingly down-regulated (*p* < 0.05), suggesting that the risk model may promote or inhibit m6A methylation by affecting the expression of these genes (**Figure 9B**).

### 3.9 NEU1-based analysis of cellular communication

After analyzing the expression of 11 genes in the risk model in TCGA-LIHC, eight genes, HLA-B, HEXB, CDK4, NAA10, B2M, HSPD1, NPM1, and NEU1, were found to be highly expressed in the tumor tissues of TCGA-LIHC (**Supplementary Figure 2**). Analysis of the differential genes between the two groups, the high-risk group and the low-risk group, revealed that the upregulation of NEU1 was the most significant (**Figure 5A**), and prognostic analyses of these 11 genes in TCGA-LIHC revealed that NEU1 had a good prognostic function (**Supplementary Figure 3**). Therefore, we concluded that NEU1 may be a key risk factor in this model.

**Figure 10:**
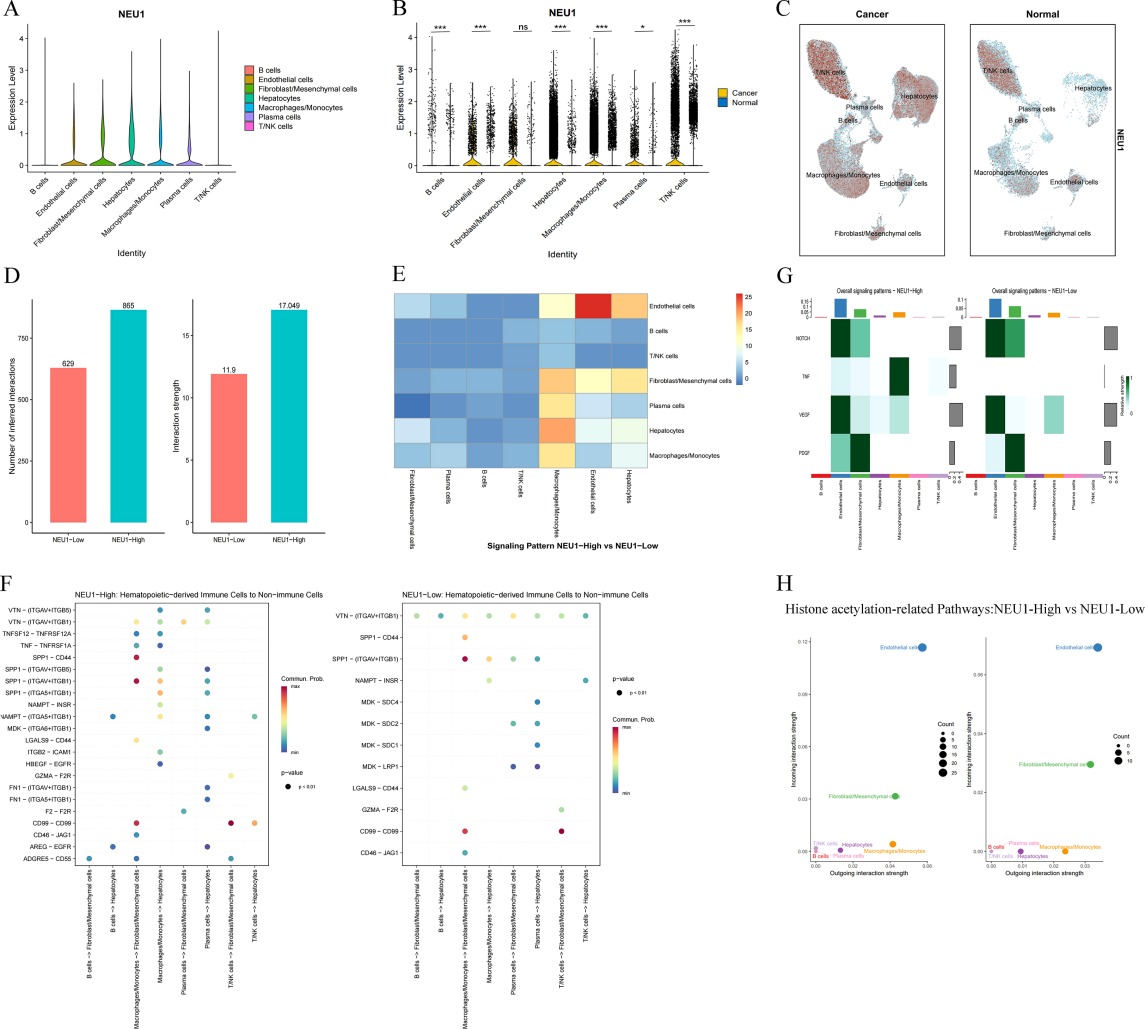
The cellular communication network of NEU1 **(A)** Expression of NEU1 on different cell clusters; **(B, C)** Comparison of NEU1 expression in tumor tissues and non-tumor tissues; **(D)** Comparison of the frequency and strength of cellular interactions in the high and low expression groups of NEU1; **(E)** Impact of changes in the expression level of NEU1 on the strength of inter-cellular communication; **(F)** Ligand-receptor interactions between different cells in the high and low expression groups of NEU1; **(G)** Intensity of histone acetylation-related pathway in different cells in the NEU1 high and low expression groups; **(H)** Comparison of the intensity of histone acetylation-related pathway interactions in different cells in the NEU1 high and low expression groups. (*p < 0.05; ***p < 0.001);.

To clarify the role of NEU1 in cellular communication, we first analyzed its expression at the single-cell level using R. NEU1 was expressed in B cells, endothelial cells, fibroblasts, mesenchymal stromal cells, hepatocytes, monocytes/macrophages, plasma cells, and T/NK cells. Compared with non-tumor tissues, NEU1 expression levels in these cell types were significantly higher in LIHC tissues (*p* < 0.05) (**Figures 10A–C**). In LIHC tissues, the NEU1 high-expression group had a higher frequency and intensity of interactions compared to the NEU1 low-expression group (**Figure 10D**). Changes in the expression level of NEU1 could lead to alterations in the intensity of communication between a variety of cells, with the highest frequency of communication between endothelial cells (**Figure 10E**). **Figure 10F** demonstrates the potential mechanisms of communication, including ligand-receptor interactions and direction of communication, between different cells in the NEU1 high-expression and NEU1 low-expression groups. For example, in the NEU1 high-expression group, monocytes/macrophages may regulate changes in fibroblast/mesenchymal cell function or phenotype through SPP1 binding to CD44 or integrins (ITGAV+ITGB1).

NOTCH signaling pathway, TNF signaling pathway, VEGF signaling pathway, and PDGF signaling pathway are common histone acetylation-related pathways, and our study showed that these four pathways were most significant in endothelial cells in both NEU1 high- and low-expression groups (**Figure 10G**). Analysis of the interaction strength of histone acetylation-related pathways in different cells revealed that the interaction strength was highest in endothelial cells and low in fibroblasts/mesenchymal stromal cells when NEU1 was highly expressed, and it remained highest in endothelial cells and increased in fibroblasts/mesenchymal stromal cells when NEU1 was low-expression (**Figure 10H**).

### 3.10 NEU1-based pseudo-timing analysis

**Figure 11:**
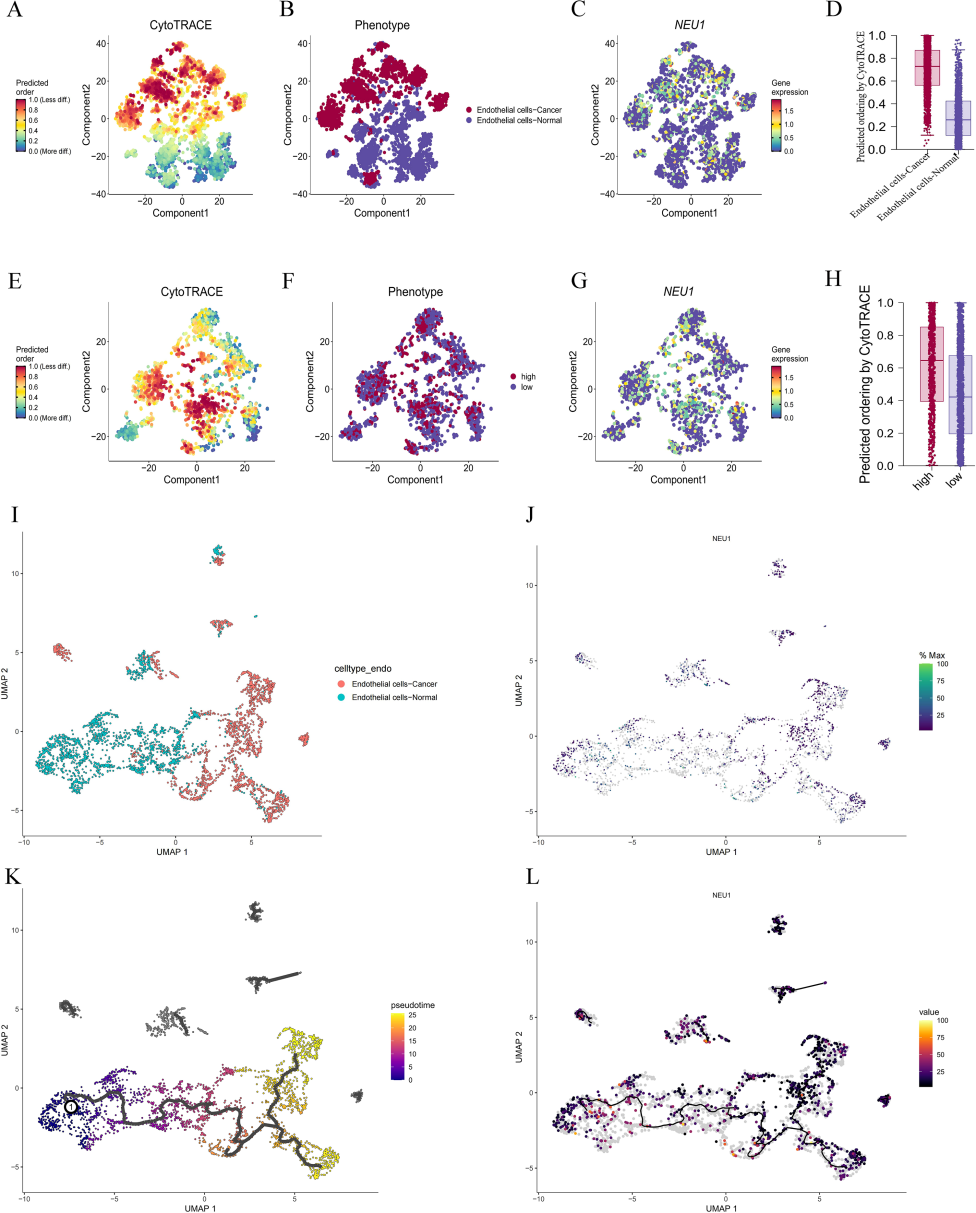
Pseudo-timing analysis of NEU1. **(A, B)** LIHC endothelial cells were less differentiated than normal endothelial cells; **(C)** NEU1 expression levels were significantly up-regulated in endothelial cells of LIHC tissues; **(D)** Endothelial cells of LIHC had higher differentiation potential.; **(E–G)** NEU1 expression levels were significantly upregulated in low-differentiated LIHC endothelial cells; **(H)** endothelial cells with high NEU1 expression have higher differentiation potential; **(I)** Monocle3 clustering analysis of heterogeneity of normal vs. LIHC endothelial cells; **(J)** Distribution of NEU1 expression in normal vs. LIHC endothelial cells; **(K)** Pseudo-temporal analysis of endothelial cell differentiation trajectories; **(L)** Dynamic changes of NEU1 expression on endothelial cell differentiation trajectories.

We used the “CytoTRACE” package to quantify cell differentiation potential and compared the CytoTRACE scores of LIHC endothelial cells with those of normal endothelial cells. The results showed that the differentiation degree of LIHC endothelial cells was lower than that of normal endothelial cells (**Figures 11A, B**). Further analysis revealed that the expression level of NEU1 was significantly up-regulated in endothelial cells of LIHC tissues (**Figure 11C**) and that the endothelial cells of LIHC had a higher differentiation potential (**Figure 11D**). Among the endothelial cell subpopulations of LIHC, the expression level of NEU1 was significantly upregulated in low-differentiated endothelial cells of LIHC, which often had higher differentiation potential, which may be an important reason why the high expression of NEU1 suggests a poor prognosis for patients with LIHC (**Figures 11E–H**).

To reveal the trajectory of endothelial cell differentiation and the potential role of NEU1 in this process, we used Monocle3 to perform the proposed time-series analysis. The analysis showed that normal endothelial cells formed highly aggregated cell clusters and exhibited high stability, whereas endothelial cells of LIHC exhibited heterogeneity that was significantly different from that of normal endothelial cells (**Figure 11I**). Further analysis revealed that the expression level of NEU1 was significantly up-regulated mainly in endothelial cells of LIHC and some normal endothelial cells, whereas it was low-expressed in most normal endothelial cells (**Figure 11J**).

Pseudo-temporal trajectory analysis revealed a stable differentiation pathway of endothelial cells along the left-to-right direction, demonstrating a sequential process of transformation of normal endothelial cells to LIHC endothelial cells (**Figure 11K**). Notably, NEU1 expression levels showed progressive up-regulation on this differentiation trajectory, suggesting that NEU1 may play a key role in the transformation of normal endothelial cells to a malignant phenotype (**Figure 11L**).

### 3.11 NEU1-based molecular docking analysis

**Figure 12:**
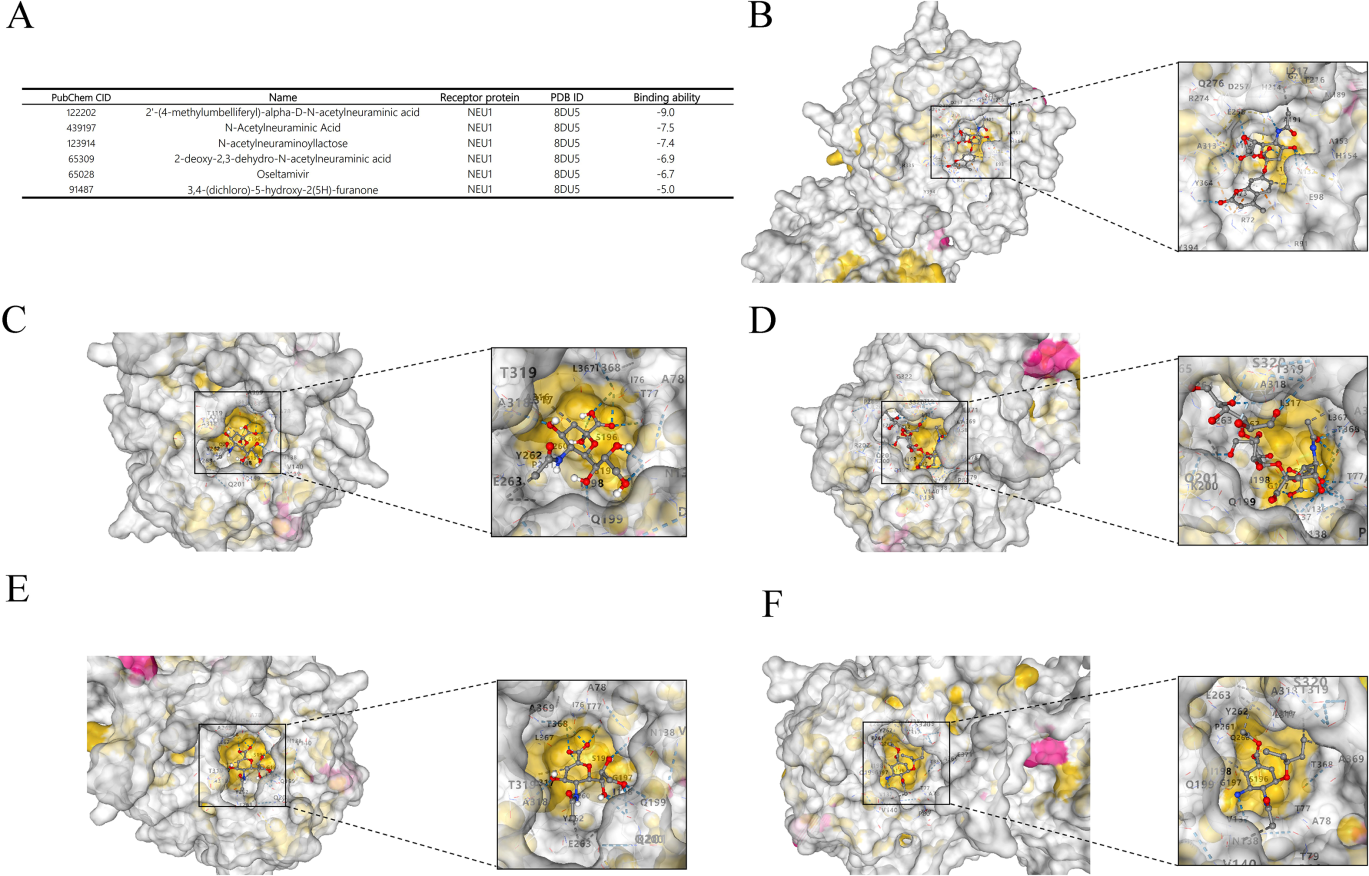
Molecular docking of NEU1 Molecular docking analysis of NEU1-targeted drugs. **(A–E)** 2’-(4-methylumbelliferyl)-α-D-N-acetylneuraminic acid, N-Acetylneuraminic acid, N-acetylneuraminolactose, 2-deoxy-2,3-dehydro-N-acetylneuraminic acid and Oseltamivir. **(F)** The results of the molecular docking analysis performed by the CB-Dock2 platform.

Ten drugs highly related to NEU1 were screened through the Coremine database, from which the active compounds with structural information available in PubChem were selected and subsequently analyzed by molecular docking in the CB-Dock2 platform. The results showed that a variety of compounds exhibited good binding activities (**Figure 12A**), among which 2’-(4-methylumbelliferyl)-α-D-N-acetylneuraminic acid, N-Acetylneuraminic acid, N acetylneuraminolactose, 2-deoxy-2,3-dehydro-N-acetylneuraminic acid, and Oseltamivir all showed binding energy values below -5 kcal/mol, suggesting that these compounds have superior binding ability to NEU1 (**Figures 12B–F**).

### 3.12 Validation of the expression of 11 genes in the risk model in LIHC samples

**Figure 13:**
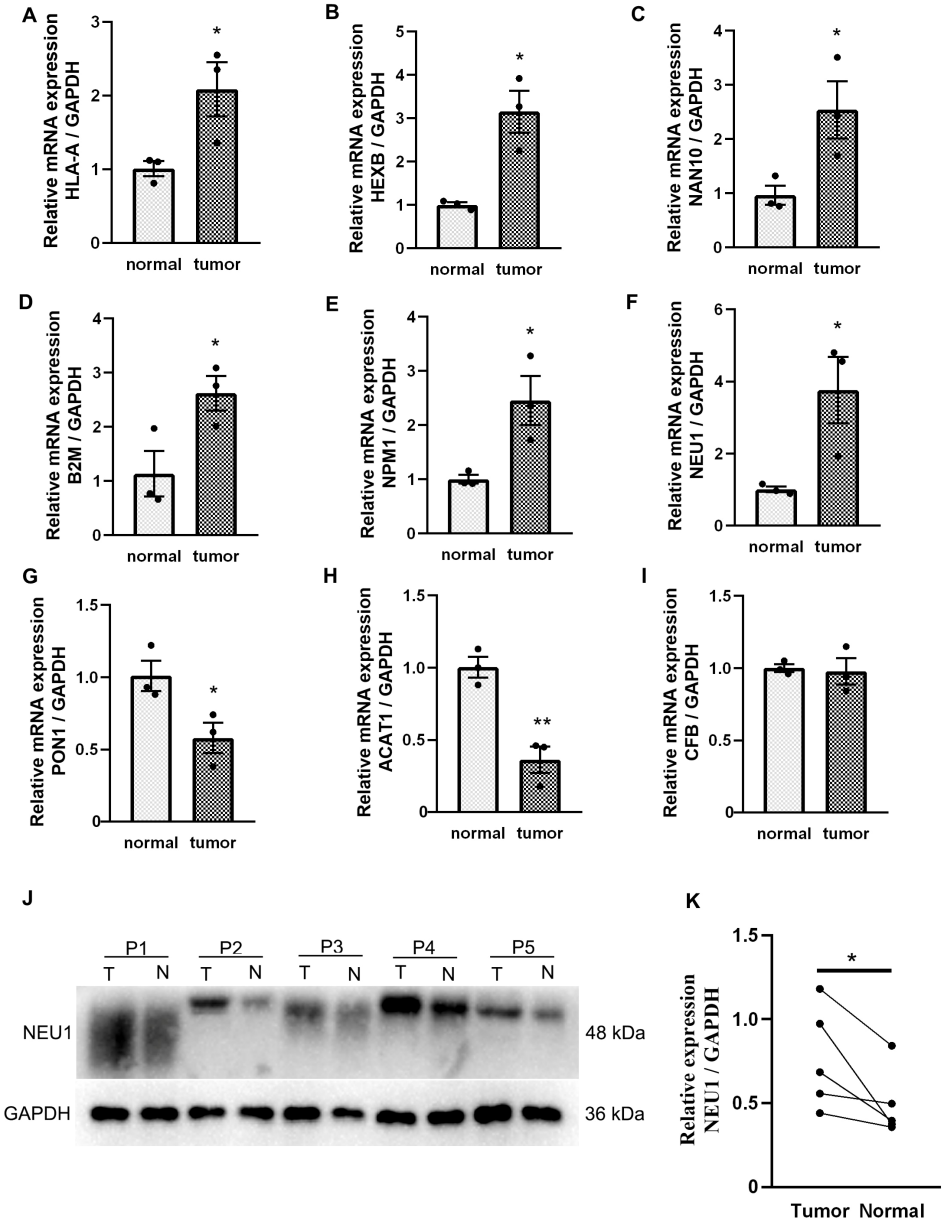
Expression of Acetylation-Associated Genes in LIHC. **(A–I)** mRNA expression of acetylation-associated genes in LIHC. n = 3; **(J, K)** NEU1 protein was detected in LIHC. n = 5, Data are presented as mean ± SEM (*p < 0.05; **p < 0.01).

We analyzed the expression of the aforementioned 11 genes in LIHC in clinical specimens using qRT-PCR. Compared with adjacent non-tumor tissues, mRNA levels of HLA-B, HEXB, NAA10, B2M, NPM1, and NEU1 were significantly upregulated in tumor tissues, while PON1 and ACAT1 were downregulated. No differential expression was observed for CFB, consistent with database analyses (**Figures 13A–I**). However, there was no statistically significant difference between CDK4 and HSPD1 in our sample (**Supplementary Figure 8**). Based on machine learning models and qPCR validation, NEU1 was selected for further investigation. Western blot analysis of LIHC in clinical specimens showed significantly elevated NEU1 protein expression in tumor tissues compared with paired adjacent non-tumor tissues (**Figures 13J, K**).

### 3.13 NEU1 knockdown inhibits proliferation, migration, and invasion of LIHC cells

**Figure 14:**
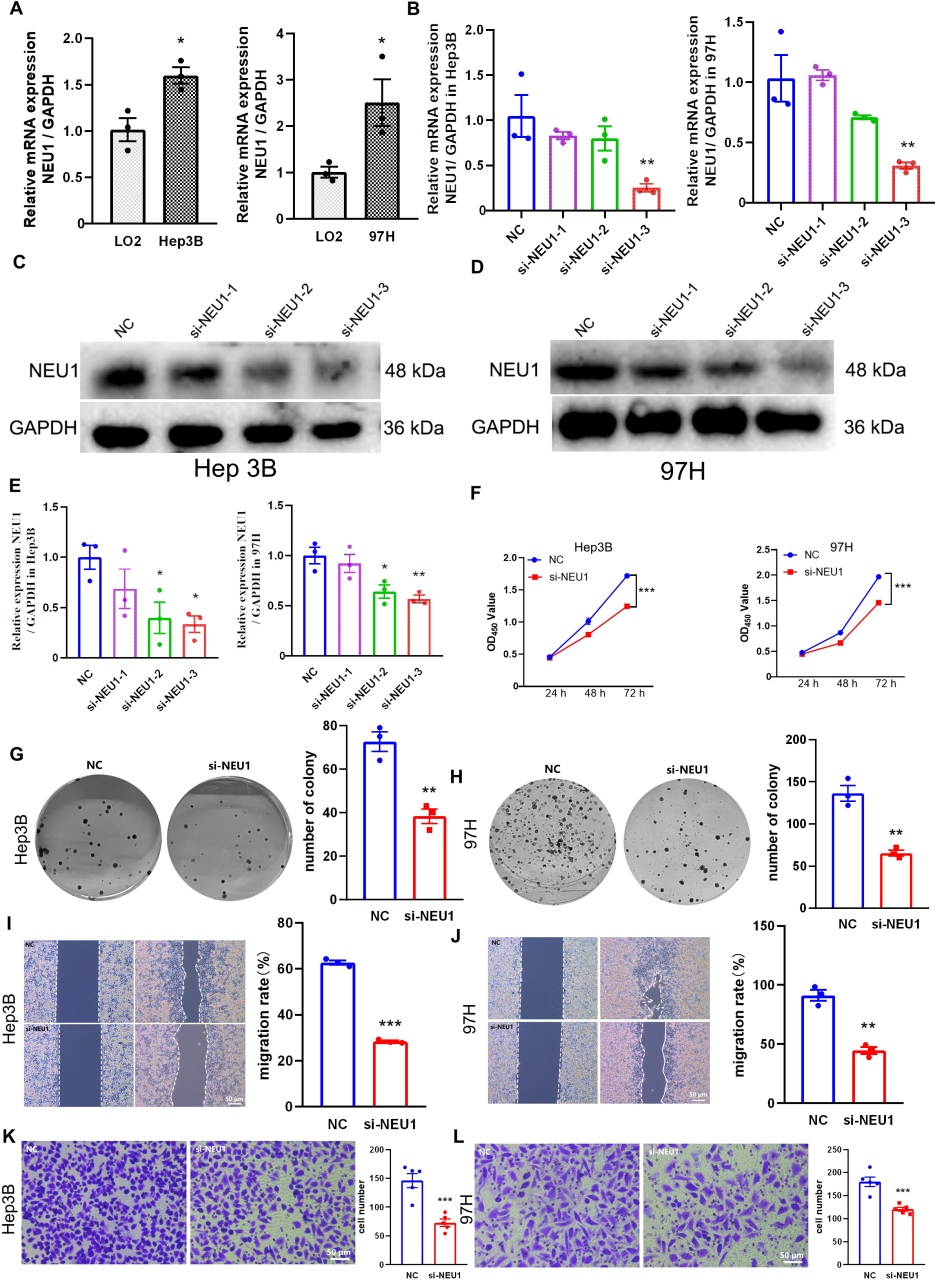
*In Vitro* Validation of the Biological functions of NEU1 in LIHC **(A)** The mRNA expression of NEU1 in Hep3B and MHCC97H vs. LO2 cell lines; **(B-E)** Verification of knockdown efficiency of NEU1 in Hep3B and MHCC97H cell lines; The biological functions of NEU1 on glioma cell lines were verified by CCK-8 **(F)**, colony formation **(G, H)**, wound healing **(I, J)** and Transwell **(K, L)** experiments. (n=3, Scale Bar = 20 μm). Data are presented as mean ± SEM. (*p < 0.05; **p < 0.01; ***p < 0.001).

Similarly, NEU1 mRNA levels were significantly higher in LIHC cell lines (Hep3B and MHCC-97H) than in the normal hepatocyte line LO2 (**Figure 14A**). To investigate the functional role of NEU1, we transfected Hep3B and MHCC-97H cells with specific siRNA targeting NEU1. We found that the si-NEU1–3 fragment exhibited robust knockdown efficacy at both the mRNA and protein levels (**Figures 14B–E**) and was therefore selected for subsequent experiments. CCK-8 assays (**Figure 14F**), clone formation assays (**Figures 14G, H**), wound healing assays(**Figures 14I, J**), and Transwell invasion assays(**Figures 14K, L**) demonstrated that NEU1 knockdown significantly suppressed proliferation, migration, and invasion in Hep3B and MHCC-97H cells.

## 4 Discussion

In this study, we successfully constructed a predictive model of LIHC risk based on histone acetylation-related genes by integrating single-cell RNA sequencing data analysis, multivariate machine learning strategy, and experimental validation, and deeply elucidated the potential molecular mechanism of NEU1 in the development of LIHC.

The study firstly, at the level of single-cell analysis, investigated the precise classification and annotation of cell populations into subpopulations of B cells, endothelial cells, fibroblasts/mesenchymal stromal cells, hepatocytes, macrophages/monocytes, plasma cells, and T/NK cells. By comparing the gene expression profiles of each cell subpopulation in normal and LIHC tissues, differentially expressed genes were identified and cross-tabulated with RNA expression profiles and histone acetylation-related genes to accurately screen out the set of histone acetylation-related differentially expressed genes. Subsequently, 101 combinations of machine learning algorithms were applied to systematically evaluate these candidate genes, and a risk prediction model consisting of 11 key genes (HLA-B, HEXB, CDK4, ACAT1, NAA10, B2M, HSPD1, NPM1, PON1, NEU1, and CFB) was finally constructed. The model demonstrated excellent predictive performance with an average C-index of 0.785, the best among all 101 machine learning algorithms, and consistently high accuracy in both the training and validation sets, which was further confirmed by Kaplan-Meier survival analysis and ROC curve analysis. Particularly noteworthy is that the model was comprehensively validated for 10 different molecular subtypes of LIHC, and showed stable prognostic predictive ability in all subtypes, which fully demonstrated its broad applicability and robustness for clinical applications.

GO and KEGG enrichment analyses of the differential genes in this model indicated that the promotion of LIHC may be achieved through key pathways such as the Small-Molecule catabolic process and Xenobiotic metabolism. In a previous study, HuiSu et al. showed that the expression level of coagulation factor F11 was significantly correlated with the infiltration of related factors and drug sensitivity in the immune environment of hepatocellular carcinoma (34). In our study, it was found that the complement and coagulation cascade response pathways may be regulated by this model, which is consistent with the findings of previous studies and further confirms the complexity of immune regulation in the microenvironment of hepatocellular carcinoma. More studies have confirmed that the lipid metabolism pathway, mTOR signaling pathway, mitochondrial oxidative phosphorylation pathway, and extracellular matrix-associated pathway play important roles in the development of LIHC (35–37).

Sun et al. showed that PA2G4 was associated with LIHC metastasis (38), and downregulation of ALDH2 expression level not only attenuated hepatic detoxification capacity but also significantly correlated with hepatic LIHC resistance to drugs and prognosis (39, 40). In the present study, GSEA analysis showed that the model may have a strong regulatory effect on the above pathways, and the scores of the model were significantly correlated with the expression levels of the core genes of these pathways (ALDH2, ADH4, SERPING1, SMS, EIF2S2, PA2G4, ACAT1, TOP2A, CLTA, and F11), further confirmed that it may promote the progression of LIHC by regulating the expression levels of these core genes, which in turn activate/inactivate the relevant pathways.

To further investigate the value of the risk model in modulating immune infiltration, the study was subjected to immunoscape analysis. Immune cell correlation showed that activated memory CD4+ T cells were positively correlated with CD8+ T cells, suggesting adaptive immune synergy, while the negative correlation between Tfh and resting memory CD4+ T cells reflected the complex regulation among immune subpopulations. The model genes HLA-B and B2M were significantly correlated with the immunity score and ESTIMATEScore, consistent with their central role as MHC-I class I molecules in antigen presentation (41). Notably, high-risk scores were associated with higher tumor purity and lower immune/stroma scores, suggesting an “immunosuppressed” or “immuno-depleted” microenvironmental phenotype, which is usually indicative of poor prognosis (42). Further analysis confirmed that the risk score correlated with the level of multiple immune cell infiltration, immune checkpoint gene expression, and key chemokines/receptors, suggesting that the risk model is capable of capturing the complex changes in immune cell recruitment, localization, and potential functional status. To evaluate the potential of clinical application, the TIDE algorithm was used to predict immune checkpoint inhibitor (ICI) responses. The results showed that the high-risk group had lower predictive scores for treatments targeting CD274 (PD-L1) and PDCD1LG2 (PD-L2), suggesting that they may be better able to benefit from anti-PD-1/PD-L1/PD-L2 therapy (43).

Based on the GDSC2 database, the study analyzed the correlation between risk scores and sensitivity to 25 clinically used chemotherapeutic agents. Patients in the high-risk group showed higher sensitivity (lower IC50 values) to Axitinib, Paclitaxel, TelomeraseInhibitorIX, and Vinblastine, which are multi-targeted tyrosine kinase inhibitors that inhibit angiogenesis, while Paclitaxel and Vinblastine, which are microtubule inhibitors, may be more effective in high-risk tumors with high proliferative properties. as microtubule inhibitors, may be more effective in high-risk tumors with high proliferative properties. This is consistent with the poor prognosis of the high-risk group (44). On the contrary, the high-risk group showed potential resistance to a variety of drugs, including DNA-damaging drugs, topoisomerase inhibitors, targeted therapeutics, and PARP inhibitors. This resistance pattern may be related to altered DNA repair mechanisms or suppression of the immune microenvironment. Notably, resistance to the mTOR inhibitor Rapamycin in the high-risk group is consistent with the association of mTOR signaling pathway activation with poor prognosis in LIHC (45). Resistance to Entinostat may reflect disturbances in epigenetic regulation in high-risk tumors. These findings provide a basis for individualized drug selection based on risk scores.

TMB analysis significantly revealed that the high-risk group exhibited a higher tumor mutational load (TMB). The high TMB may stem from the functional deficiencies of DNA repair genes, which may also explain the resistance to DNA-damaging drugs in the high-risk group. The relevant gene mutations in the low-risk group may activate pathways that favor anti-tumor immunity, while the relevant gene mutations in the high-risk group may activate pathways that promote immune escape.

The synergistic role of ferroptosis and m6A methylation abnormalities in LIHC may be an important molecular basis for the poor prognosis of high-risk patients. For example, m6A modifications may affect the expression of key genes for ferroptosis, forming a complex regulatory network (46). In our study, we found that the risk model score was significantly correlated with the expression of most ferroptosis and m6A methylation-related genes, suggesting that it may play an important role in promoting the progression of LIHC through the regulation of ferroptosis and m6A methylation. However, the specific mechanism needs to be further explored.

Cell communication analysis is an analytical method to study how different cell types communicate and regulate each other through signaling molecules such as ligands and receptors. It is important in revealing the mechanisms of cellular interactions and understanding how tissues and organs function in a coordinated manner (47).

The cellular communication analysis in this study indicated that high NEU1 expression may promote the activation of multiple inflammatory factors and matrix remodeling in LIHC by enhancing SPP1-mediated communication between monocytes/macrophages and fibroblasts/mesenchymal stromal cells. This mechanism may be a key factor in the formation of the immunosuppressive microenvironment in LIHC.

Quantification of cell differentiation potential based on the CytoTRACE algorithm suggested that NEU1 may promote tumor neovascularization and progression by maintaining the low differentiation state of endothelial cells.Monocle3 algorithm proposed temporal sequential analysis revealed the differentiation pathway of endothelial cells along a specific direction, demonstrating a sequential process of the transformation of normal endothelial cells to LIHC endothelial cells, and the expression level of NEU1 The expression level of NEU1 was progressively up-regulated on this differentiation trajectory, suggesting that NEU1 may play a key driving role in the transformation of normal endothelial cells to malignant phenotype. This may be an important mechanism by which high expression of NEU1 contributes to the poor prognosis of LIHC patients. Future studies will focus on verifying the causal relationship between NEU1 in endothelial cell differentiation and malignant transformation and exploring its potential as an anti-angiogenic target. Meanwhile, abnormal endothelial cell differentiation may also provide new ideas for early diagnosis and prognosis of LIHC.

Our study identifies NEU1 as a pivotal node linking sialometabolism to histone acetylation. As a lysosomal sialidase, NEU1 hydrolysis of sialoglycoconjugates modulates cellular pools of free sialic acid and UDP-GlcNAc, which are precursors for nucleotide sugar biosynthesis (48). This process indirectly regulates the availability of acetyl-CoA—a central metabolite and essential cofactor for histone acetyltransferases (HATs). The observed NEU1-driven activation of histone acetylation-related pathways (e.g., NOTCH, VEGF) may thus stem from its ability to fuel epigenetic machinery via metabolic rewiring. Notably, the NEU1 inhibitor Oseltamivir (identified by molecular docking) has been repurposed for cancer therapy in recent studies targeting sialylation-dependent immune evasion (49). This further underscores NEU1’s druggability in modulating the epigenetic landscape of LIHC.

To further corroborate the value of the histone acetylation-related risk model in LIHC, we first validated the expression of 11 genes in the risk model in LIHC clinical samples using qRT-PCR experiments and the expression of NEU1 using WB experiments. The results showed that, consistent with our previous analysis, six genes, including NEU1, were up-regulated and ACAT1 and PON1 were down-regulated in LIHC tissues. Compared with normal tissues, there was no difference in the expression of CDK4 and HSPD1 in LIHC tissues, which might be the reason that the sample size was too small, and we will further expand the sample size for validation in the follow-up study. We performed relevant experiments *in vitro* and *in vivo* to clarify the role of NEU1 in LIHC progression, respectively. The proliferation, invasion, and migration of Hep3B and MHCC-97H cells were attenuated after NEU1 knockdown. These results confirmed the promotional role of NEU1 as a key factor in histone acetylation-associated risk models for LIHC development.

This study clarifies the important role of the histone acetylation-associated risk model in LIHC based on multi-omics data, clinical samples, and experiments, which has the potential to become yet another novel diagnostic and prognostic model, providing a new target for precision treatment of LIHC.

Despite the progress of our study, there are still some shortcomings, such as the downstream molecules and signaling pathways of the risk model are not fully understood, and more experimental basis is needed for the regulatory role of NEU1 on LIHC progression. We will explore this more deeply in subsequent studies.

## Data Availability

The datasets presented in this study can be found in online repositories. The names of the repository/repositories and accession number(s) can be found in the article/**Supplementary Material**.

## Supplementary material

Supplementary Figure 1 Basis for cell type annotation.

Supplementary Figure 2 Expression of 11 genes in the histone acetylation-related risk model in TCGA-LIHC.

Supplementary Figure 3 Prognostic value of 11 genes in the histone acetylation-related risk model in TCGA-LIHC **(A)** ACAT1; **(B)** B2M; **(C)** CDK4; **(D)** CFB; **(E)** HEXB; **(F)** HLA – B; **(G)** HSPD1; **(H)** NAA10; **(I)** NEU1; **(J)** NPM1; **(K)** PON1.

Supplementary Figure 4 Independent validation of the model and AUC assessment **(A, B)** Independent validation of the model in OEP000321 cohort; **(C, E)** AUC assessment.

Supplementary Figure 5 Prognostic performance of the risk signature in the ICGC-LIRI-JP validation cohort and its clinical subgroups. Kaplan-Meier curves for Overall Survival (OS) are shown for low-risk versus high risk patients in the entire cohort and within subgroups stratified by different clinical variables. P-values were determined using the log-rank test. The analyses are shown for **(A)** the entire cohort, and for subgroups based on: **(B)** Age > median, **(C)** Age ≤ median, **(D)** Male patients, **(E)** Female patients, **(F)** HBV-infected patients, **(G)** HCV- infected patients, (1166 **(H)**) TNM stage I-II, **(I)** TNM stage III-IV, **(J)** Fibrosis stage F0-F2, and **(K)** Fibrosis stage F3-F4.

Supplementary Figure 6 Prognostic performance of the risk signature in the entire GSE124751 Ncohort and within clinical subgroups. Kaplan-Meier curves comparing Overall Survival (OS) and Disease-Free Survival (DFS) between the high-risk and low-risk groups. Analyses were performed for the entire cohort and for subgroups stratified by age (cutoff at 60 years) and Tumor Mutational Burden (TMB, cutoff at the median). P-values were calculated using the log-rank test. Overall Survival analysis in **(A)** the entire cohort, and in subgroups stratified by **(B)** Age > 60, **(C)** Age ≤ 60, **(D)** High TMB, and **(E)** Low TMB. Disease-Free Survival analysis in **(F)** the entire cohort, and in subgroups stratified by **(G)** Age > 60, **(H)** Age ≤ 60, **(I)** High TMB, and **(J)** Low TMB.

Supplementary Figure 7 Comparative analysis of the mutational landscape across three key hepatocellular carcinoma (HCC) cohorts from cBioPortal. The molecular profiles of the primary TCGA-LIHC cohort were compared with two independent cohorts (MSK 2024, CLCA 2024) to assess molecular heterogeneity. **(A)** An Oncoprint visualizing specific alterations in a panel of key genes (including HLA-B, NEU1, and CDK4) for individual patients within each cohort. Each column represents a patient, and each row a gene. Colors denote the type of alteration. **(B)** A stacked bar plot summarizing the frequency of major alteration types (e.g., amplification, deep deletion, mutation) across the three cohorts, highlighting the dominant genomic alteration in each study. **(C)** Boxplot showing the distribution of mutation counts corresponding to different Copy Number Alteration (CNA) types (e.g., amplification, gain, diploid, shallow deletion) for the NEU1 gene in the MSK-IMPACT cohort.

Supplementary Figure 8 Clinical sample validation of CDK4 and HSDP1 mRNA expression levels.

## References

[1] Bray F Laversanne M Sung H Ferlay J Siegel RL Soerjomataram I . Global cancer statistics 2022: GLOBOCAN estimates of incidence and mortality worldwide for 36 cancers in 185 countries. CA Cancer J Clin. (2024) 74:229–63. doi: 10.3322/caac.21834, PMID: 38572751

[2] Peng Z Fan W Zhu B Wang G Sun J Xiao C . Lenvatinib combined with transarterial chemoembolization as first-line treatment for advanced hepatocellular carcinoma: A phase III, randomized clinical trial (LAUNCH). J Clin Oncol. (2023) 41:117–27. doi: 10.1200/JCO.22.00392, PMID: 35921605

[3] Zhang Y Huang G Wang Y Liang L Peng B Fan W . Is salvage liver resection necessary for initially unresectable hepatocellular carcinoma patients downstaged by transarterial chemoembolization? Ten years of experience. Oncologist. (2016) 21:1442–9. doi: 10.1634/theoncologist.2016-0094, PMID: 27486202 PMC5153353

[4] Bruix J Qin S Merle P Granito A Huang YH Bodoky G . Regorafenib for patients with hepatocellular carcinoma who progressed on sorafenib treatment (RESORCE): a randomised, double-blind, placebo-controlled, phase 3 trial. Lancet. (2017) 389:56–66. doi: 10.1016/S0140-6736(16)32453-9, PMID: 27932229

[5] Lok AS Sterling RK Everhart JE Wright EC Hoefs JC Di Bisceglie AM . Des-gamma-carboxy prothrombin and alpha-fetoprotein as biomarkers for the early detection of hepatocellular carcinoma. Gastroenterology. (2010) 138:493–502. doi: 10.1053/j.gastro.2009.10.031, PMID: 19852963 PMC2819612

[6] Lu Y Yang A Quan C Pan Y Zhang H Li Y . A single-cell atlas of the multicellular ecosystem of primary and metastatic hepatocellular carcinoma. Nat Commun. (2022) 13:4594. doi: 10.1038/s41467-022-32283-3, PMID: 35933472 PMC9357016

[7] Meng Y Zhao Q An L Jiao S Li R Sang Y . A TNFR2-hnRNPK axis promotes primary liver cancer development via activation of YAP signaling in hepatic progenitor cells. Cancer Res. (2021) 81:3036–50. doi: 10.1158/0008-5472.CAN-20-3175, PMID: 33619115

[8] Hao Y Stuart T Kowalski MH Choudhary S Hoffman P Hartman A . Dictionary learning for integrative, multimodal and scalable single-cell analysis. Nat Biotechnol. (2024) 42:293–304. doi: 10.1038/s41587-023-01767-y, PMID: 37231261 PMC10928517

[9] Ritchie ME Phipson B Wu D Hu Y Law CW Shi W . limma powers differential expression analyses for RNA-sequencing and microarray studies. Nucleic Acids Res. (2015) 43:e47. doi: 10.1093/nar/gkv007, PMID: 25605792 PMC4402510

[10] Cao J Yan Q . Cancer epigenetics, tumor immunity, and immunotherapy. Trends Cancer. (2020) 6:580–92. doi: 10.1016/j.trecan.2020.02.003, PMID: 32610068 PMC7330177

[11] Yuan X Rosen JM . Histone acetylation modulators in breast cancer. Breast Cancer Res. (2025) 27:49. doi: 10.1186/s13058-025-02006-9, PMID: 40165290 PMC11959873

[12] Suraweera A O'Byrne KJ Richard DJ . Epigenetic drugs in cancer therapy. Cancer Metastasis Rev. (2025) 44:37. doi: 10.1007/s10555-025-10253-7, PMID: 40011240 PMC11865116

[13] Kim JJ Lee SY Choi JH Woo HG Xhemalce B Miller KM . PCAF-mediated histone acetylation promotes replication fork degradation by MRE11 and EXO1 in BRCA-deficient cells. Mol Cell. (2020) 80:327–344.e8. doi: 10.1016/j.molcel.2020.08.018, PMID: 32966758 PMC7572766

[14] Chen P Hsu WH Chang A Tan Z Lan Z Zhou A . Circadian regulator CLOCK recruits immune-suppressive microglia into the GBM tumor microenvironment. Cancer Discov. (2020) 10:371–81. doi: 10.1158/2159-8290.CD-19-0400, PMID: 31919052 PMC7058515

[15] Guo X Huang G Qiu D He H Niu X Guo Z . RPS6KA1 is a histone acetylation-related oncoprotein in acute myeloid leukemia which is targeted by afzelin. BMC Cancer. (2024) 24:1189. doi: 10.1186/s12885-024-12886-3, PMID: 39333927 PMC11438311

[16] Liu H Zhang W Zhang Y Adegboro AA Fasoranti DO Dai L . Mime: A flexible machine-learning framework to construct and visualize models for clinical characteristics prediction and feature selection. Comput Struct Biotechnol J. (2024) 23:2798–810. doi: 10.1016/j.csbj.2024.06.035, PMID: 39055398 PMC11269309

[17] Blanche P Dartigues JF Jacqmin-Gadda H . Estimating and comparing time-dependent areas under receiver operating characteristic curves for censored event times with competing risks. Stat Med. (2013) 32:5381–97. doi: 10.1002/sim.5958, PMID: 24027076

[18] Robin X Turck N Hainard A Tiberti N Lisacek F Sanchez JC . pROC: an open-source package for R and S+ to analyze and compare ROC curves. BMC Bioinf. (2011) 12:77. doi: 10.1186/1471-2105-12-77, PMID: 21414208 PMC3068975

[19] Wu T Hu E Xu S Chen M Guo P Dai Z . clusterProfiler 4.0: A universal enrichment tool for interpreting omics data. Innovation (Camb). (2021) 2:100141. doi: 10.1016/j.xinn.2021.100141, PMID: 34557778 PMC8454663

[20] Becht E Giraldo NA Lacroix L Buttard B Elarouci N Petitprez F . Estimating the population abundance of tissue-infiltrating immune and stromal cell populations using gene expression. Genome Biol. (2016) 17:218. doi: 10.1186/s13059-016-1070-5, PMID: 27765066 PMC5073889

[21] Newman AM Liu CL Green MR Gentles AJ Feng W Xu Y . Robust enumeration of cell subsets from tissue expression profiles. Nat Methods. (2015) 12:453–7. doi: 10.1038/nmeth.3337, PMID: 25822800 PMC4739640

[22] Aran D Hu Z Butte AJ . xCell: digitally portraying the tissue cellular heterogeneity landscape. Genome Biol. (2017) 18:220. doi: 10.1186/s13059-017-1349-1, PMID: 29141660 PMC5688663

[23] Maeser D Gruener RF Huang RS . oncoPredict: an R package for predicting *in vivo* or cancer patient drug response and biomarkers from cell line screening data. Brief Bioinform. (2021) 22:bbab260. doi: 10.1093/bib/bbab260, PMID: 34260682 PMC8574972

[24] Mayakonda A Lin DC Assenov Y Plass C Koeffler HP . Maftools: efficient and comprehensive analysis of somatic variants in cancer. Genome Res. (2018) 28:1747–56. doi: 10.1101/gr.239244.118, PMID: 30341162 PMC6211645

[25] Liu Z Zhao Q Zuo ZX Yuan SQ Yu K Zhang Q . Systematic analysis of the aberrances and functional implications of ferroptosis in cancer. iScience. (2020) 23:101302. doi: 10.1016/j.isci.2020.101302, PMID: 32629423 PMC7334617

[26] Li Z Li Y Shen L Shen L Li N . Molecular characterization, clinical relevance and immune feature of m7G regulator genes across 33 cancer types. Front Genet. (2022) 13:981567. doi: 10.3389/fgene.2022.981567, PMID: 36092891 PMC9453236

[27] Jin S Guerrero-Juarez CF Zhang L Chang I Ramos R Kuan CH . Inference and analysis of cell-cell communication using CellChat. Nat Commun. (2021) 12:1088. doi: 10.1038/s41467-021-21246-9, PMID: 33597522 PMC7889871

[28] Gulati GS Sikandar SS Wesche DJ Manjunath A Bharadwaj A Berger MJ . Single-cell transcriptional diversity is a hallmark of developmental potential. Science. (2020) 367:405–11. doi: 10.1126/science.aax0249, PMID: 31974247 PMC7694873

[29] Qiu X Hill A Packer J Lin D Ma YA Trapnell C . Single-cell mRNA quantification and differential analysis with Census. Nat Methods. (2017) 14:309–15. doi: 10.1038/nmeth.4150, PMID: 28114287 PMC5330805

[30] Liu Y Cao Y . Protein-ligand blind docking using CB-dock2. Methods Mol Biol. (2024) 2714:113–25. doi: 10.1007/978-1-0716-3441-7_6, PMID: 37676595

[31] Yang X Liu Y Gan J Xiao ZX Cao Y . FitDock: protein-ligand docking by template fitting. Brief Bioinform. (2022) 23:bbac087. doi: 10.1093/bib/bbac087, PMID: 35289358

[32] Su H Chen Y Wang W . Novel prognostic model of complement and coagulation cascade-related genes correlates with immune environment and drug sensitivity in hepatocellular carcinoma. Heliyon. (2024) 10:e38230. doi: 10.1016/j.heliyon.2024.e38230, PMID: 39391504 PMC11466567

[33] Liu Q Zhang X Qi J Tian X Dovjak E Zhang J . Comprehensive profiling of lipid metabolic reprogramming expands precision medicine for LIHC. Hepatology. (2025) 81:1164–80. doi: 10.1097/HEP.0000000000000962, PMID: 38899975 PMC11902616

[34] Guri Y Colombi M Dazert E Hindupur SK Roszik J Moes S . mTORC2 promotes tumorigenesis via lipid synthesis. Cancer Cell. (2017) 32:807–823.e12. doi: 10.1016/j.ccell.2017.11.011, PMID: 29232555

[35] Kudo Y Sugimoto M Arias E Kasashima H Cordes T Linares JF . PKCλ/ι Loss induces autophagy, oxidative phosphorylation, and NRF2 to promote liver cancer progression. Cancer Cell. (2020) 38:247–262.e11. doi: 10.1016/j.ccell.2020.05.018, PMID: 32589943 PMC7423690

[36] Sun S Liu Y Zhou M Wen J Xue L Han S . PA2G4 promotes the metastasis of hepatocellular carcinoma by stabilizing FYN mRNA in a YTHDF2-dependent manner. Cell Biosci. (2022) 12:55. doi: 10.1186/s13578-022-00788-5, PMID: 35526051 PMC9080163

[37] Seo W Gao Y He Y Sun J Xu H Feng D . ALDH2 deficiency promotes alcohol-associated liver cancer by activating oncogenic pathways via oxidized DNA-enriched extracellular vesicles. J Hepatol. (2019) 71:1000–11. doi: 10.1016/j.jhep.2019.06.018, PMID: 31279903 PMC6801025

[38] Tsai MC Yang SS Lin CC Wang WL Hsu YC Chen YS . Association of heavy alcohol intake and ALDH2 rs671 polymorphism with hepatocellular carcinoma and mortality in patients with hepatitis B virus-related cirrhosis. JAMA Netw Open. (2022) 5:e2223511. doi: 10.1001/jamanetworkopen.2022.23511, PMID: 35877121 PMC9315423

[39] Beck JD Diken M Suchan M Streuber M Diken E Kolb L . Long-lasting mRNA-encoded interleukin-2 restores CD8+ T cell neoantigen immunity in MHC class I-deficient cancers. Cancer Cell. (2024) 42:1467–70. doi: 10.1016/j.ccell.2024.07.010, PMID: 39137730

[40] Kemi N Eskuri M Herva A Leppänen J Huhta H Helminen O . Tumour-stroma ratio and prognosis in gastric adenocarcinoma. Br J Cancer. (2018) 119:435–9. doi: 10.1038/s41416-018-0202-y, PMID: 30057407 PMC6133938

[41] Baghy K Ladányi A Reszegi A Kovalszky I . Insights into the tumor microenvironment-components, functions and therapeutics. Int J Mol Sci. (2023) 24:17536. doi: 10.3390/ijms242417536, PMID: 38139365 PMC10743805

[42] Fukumura D Kloepper J Amoozgar Z Duda DG Jain RK . Enhancing cancer immunotherapy using antiangiogenics: opportunities and challenges. Nat Rev Clin Oncol. (2018) 15:325–40. doi: 10.1038/nrclinonc.2018.29, PMID: 29508855 PMC5921900

[43] Arriola Apelo SI Lamming DW . Rapamycin: an inhibiTOR of aging emerges from the soil of easter island. J Gerontol A Biol Sci Med Sci. (2016) 71:841–9. doi: 10.1093/gerona/glw090, PMID: 27208895 PMC4906330

[44] Jiang X Liu B Nie Z Duan L Xiong Q Jin Z . The role of m6A modification in the biological functions and diseases. Signal Transduct Target Ther. (2021) 6:74. doi: 10.1038/s41392-020-00450-x, PMID: 33611339 PMC7897327

[45] Sathe A Mason K Grimes SM Zhou Z Lau BT Bai X . Colorectal cancer metastases in the liver establish immunosuppressive spatial networking between tumor-associated SPP1+ Macrophages and fibroblasts. Clin Cancer Res. (2023) 29:244–60. doi: 10.1158/1078-0432.CCR-22-2041, PMID: 36239989 PMC9811165

[46] Itoh K Tsukimoto J . Lysosomal sialidase NEU1, its intracellular properties, deficiency, and use as a therapeutic agent. Glycoconj J. (2023) 40:611–9. doi: 10.1007/s10719-023-10135-6, PMID: 38147151

[47] Chen Y Han P Zhu H Zhang W Ma X He Y . New use of an old drug: mechanism of oseltamivir phosphate inhibiting liver cancer through regulation of lipophagy via NEU1. Front Pharmacol. (2025) 16:1556661. doi: 10.3389/fphar.2025.1556661, PMID: 40196362 PMC11973263

[48] Kiełbowski K Szwedkowicz A Plewa P Bakinowska E Becht R Pawlik A . Anticancer properties of histone deacetylase inhibitors - what is their potential? Expert Rev Anticancer Ther. (2025) 25:105–20. doi: 10.1080/14737140.2025.2452338, PMID: 39791841

[49] Liu Y Yang X Gan J Chen S Xiao ZX Cao Y . CB-Dock2: improved protein-ligand blind docking by integrating cavity detection, docking and homologous template fitting. Nucleic Acids Res. (2022) 50:W159–64. doi: 10.1093/nar/gkac394, PMID: 35609983 PMC9252749

