# Supplementary figures and images for: Integration of single-cell sequencing, transcriptome sequencing, and machine learning for constructing and validating histone acetylation-related prognostic risk models in hepatocellular carcinoma
Source: Front Immunol. 2026 Jan 23;17:1624883. doi: 10.3389/fimmu.2026.1624883 (PMC12876249; doi:10.3389/fimmu.2026.1624883)

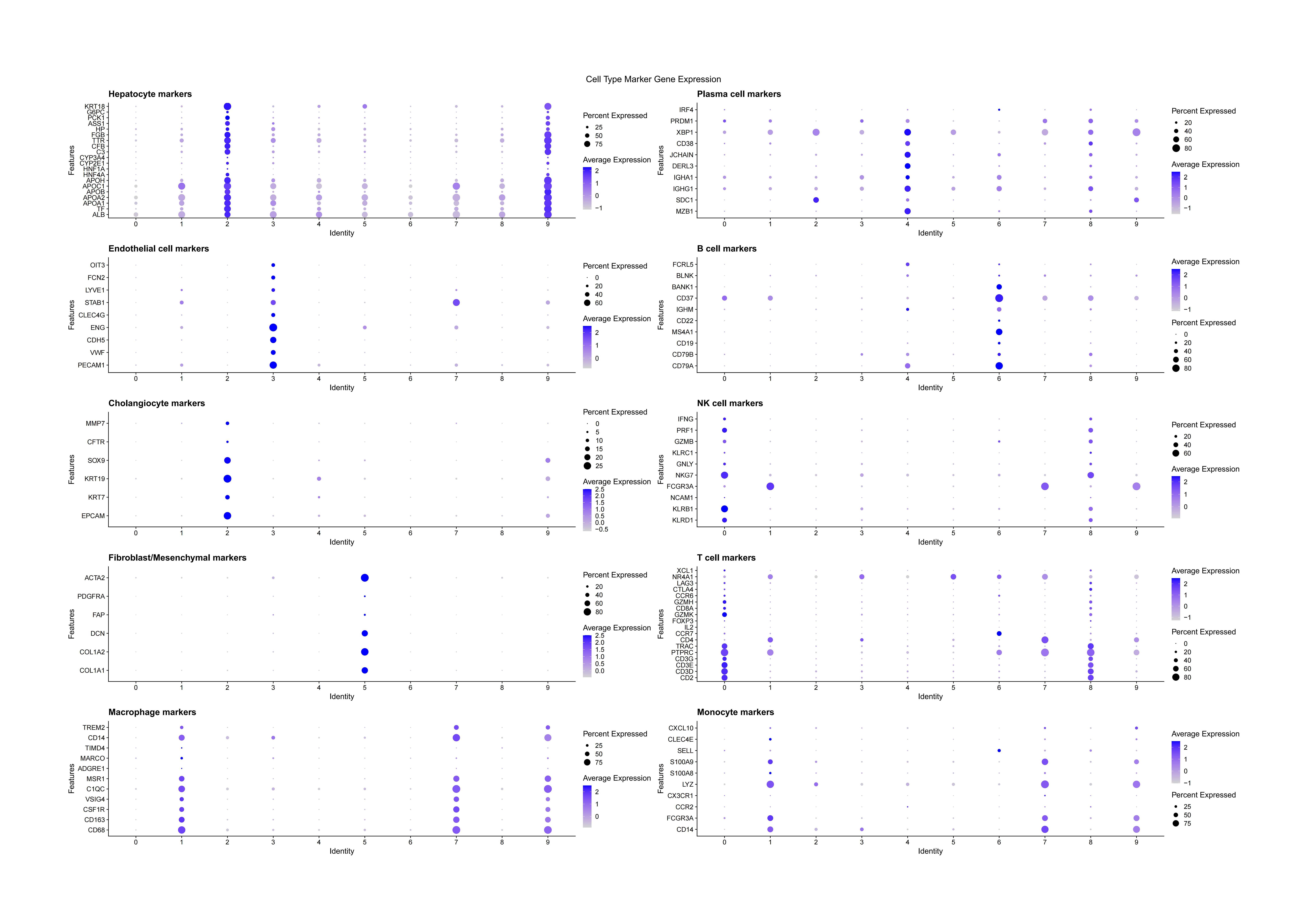

Supplement: Supplementary Figure 1 — Basis for cell type annotation. [file Image1.jpg]

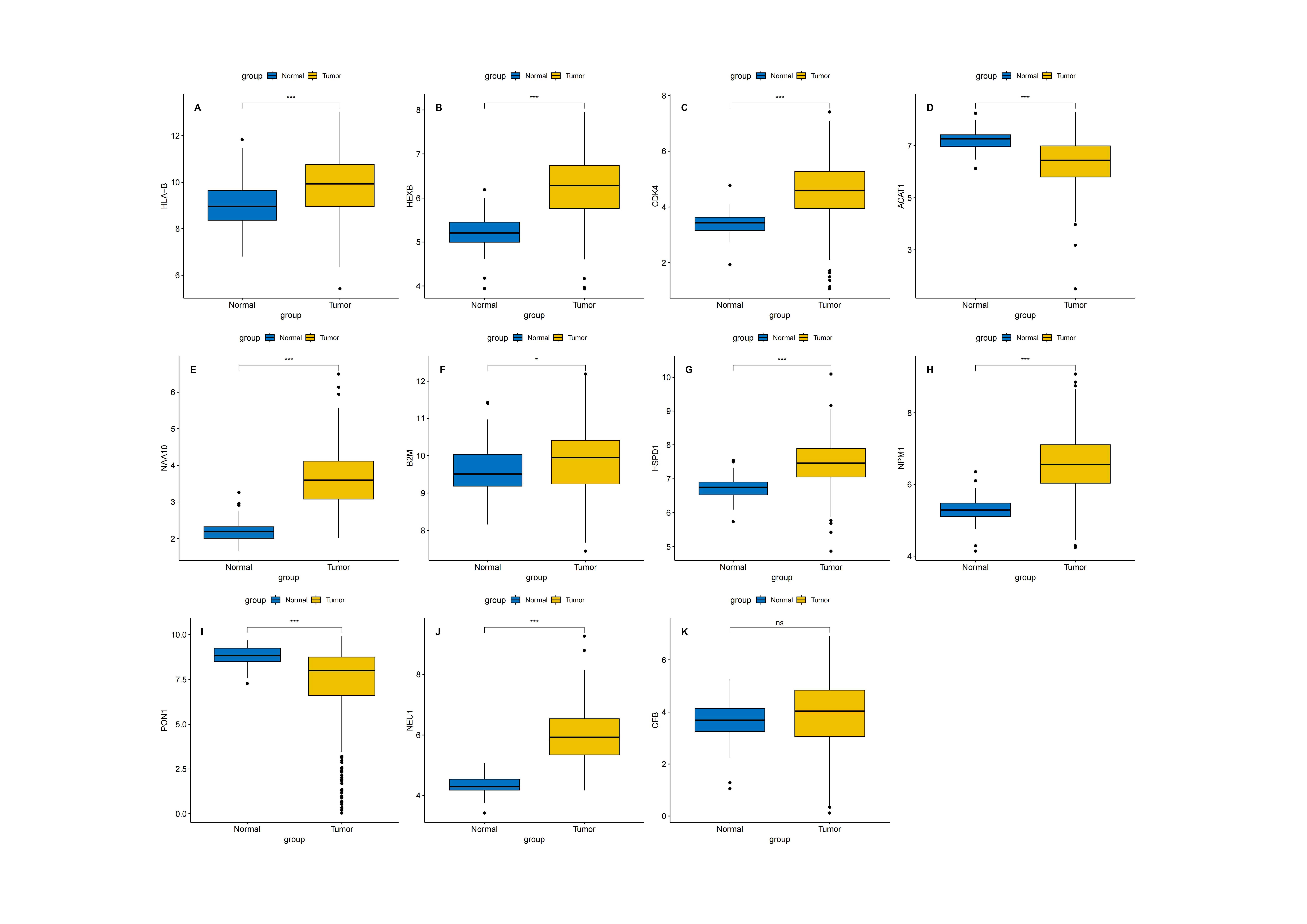

Supplement: Supplementary Figure 2 — Expression of 11 genes in the histone acetylation-related risk model in TCGA-LIHC. [file Image2.jpg]

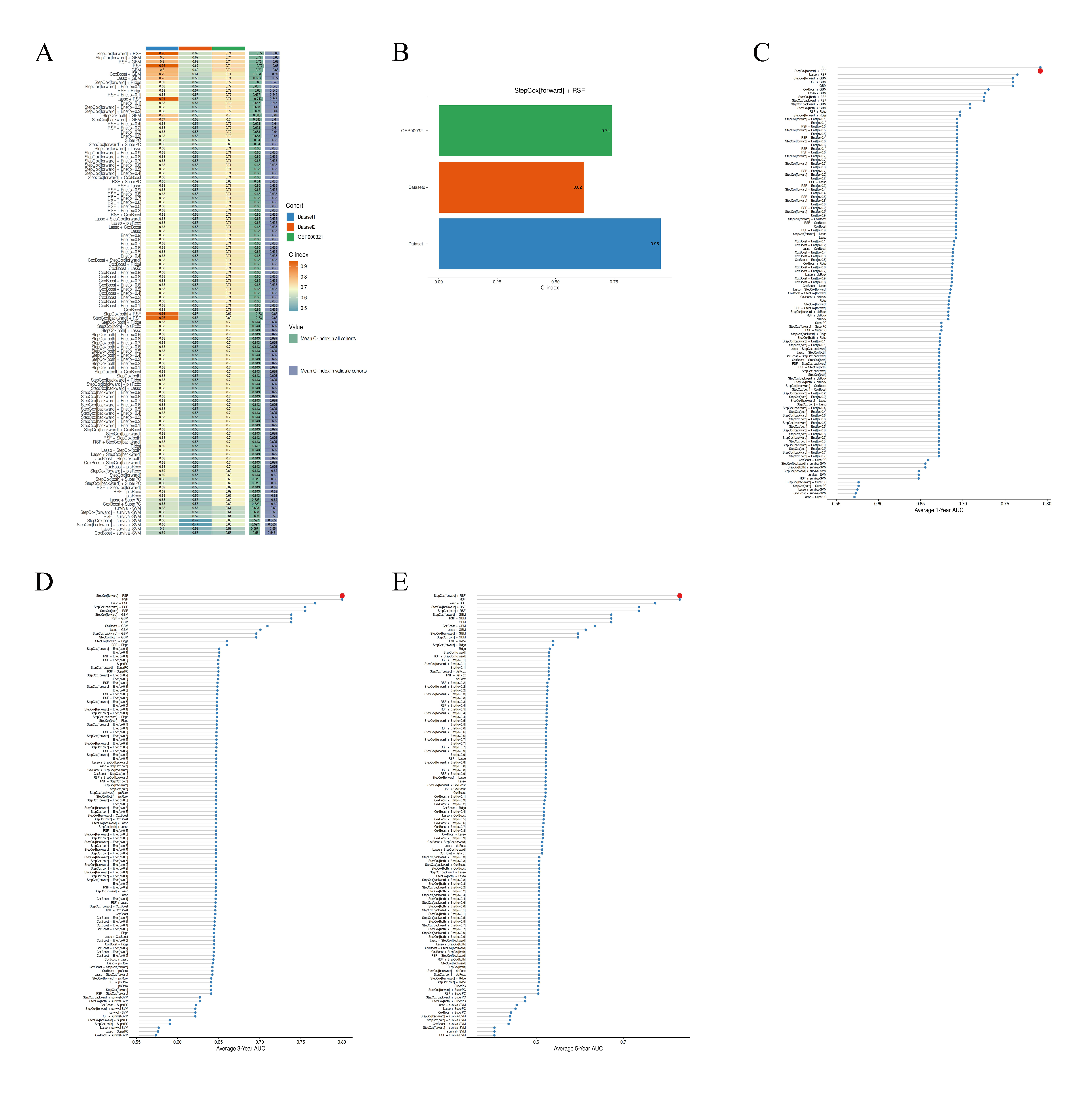

Supplement: Supplementary Figure 4 — Independent validation of the model and AUC assessment (A, B) Independent validation of the model in OEP000321 cohort; (C, E) AUC assessment. [file Image4.tif]

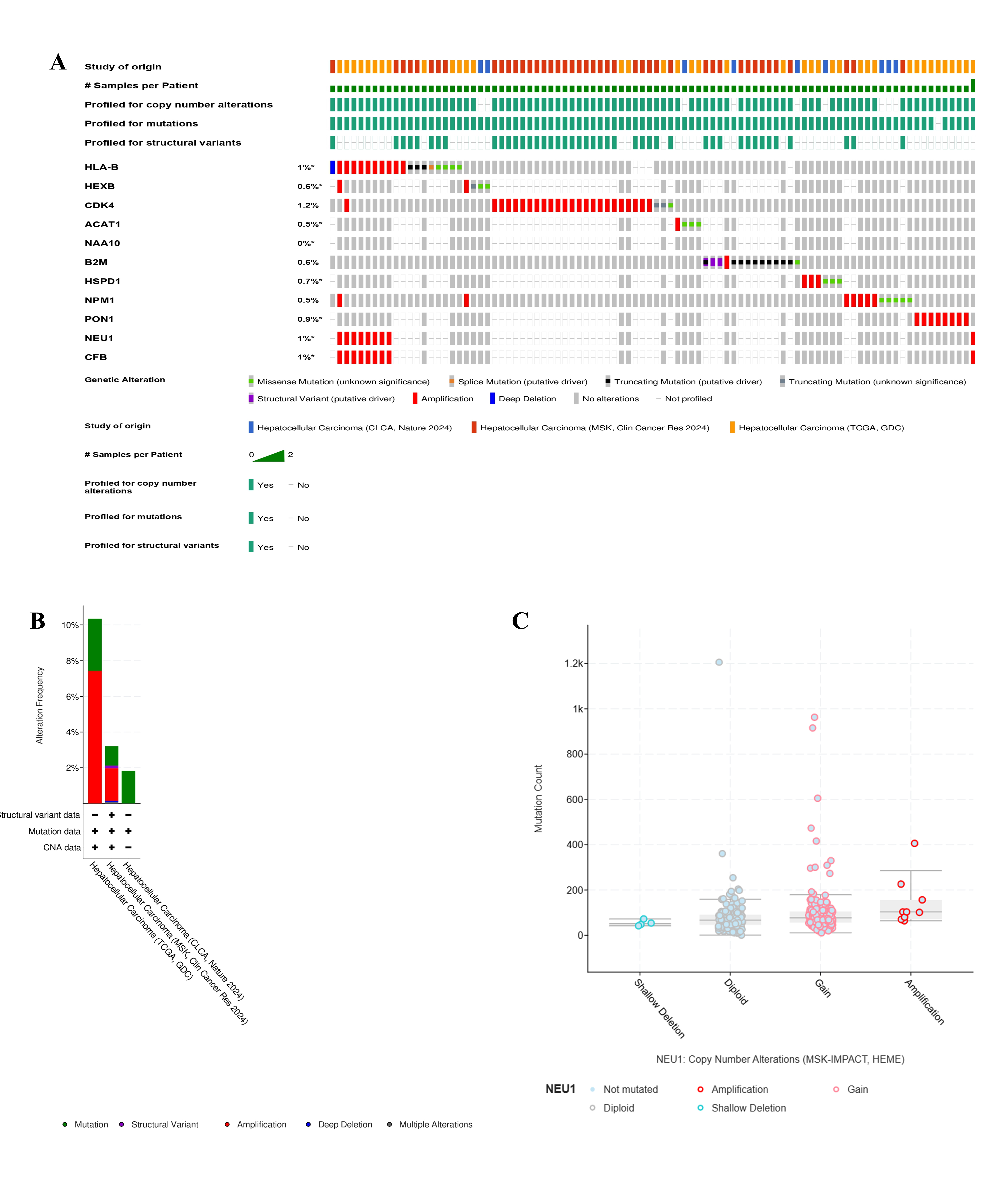

Supplement: Supplementary Figure 7 — Comparative analysis of the mutational landscape across three key hepatocellular carcinoma (HCC) cohorts from cBioPortal. The molecular profiles of the primary TCGA-LIHC cohort were compared with two independent cohorts (MSK 2024, CLCA 2024) to assess molecular heterogeneity. (A) An Oncoprint visualizing specific alterations in a panel of key genes (including HLA-B, NEU1, and CDK4) for individual patients within each cohort. Each column represents a patient, and each row a gene. Colors denote the type of alteration. (B) A stacked bar plot summarizing the frequency of major alteration types (e.g., amplification, deep deletion, mutation) across the three cohorts, highlighting the dominant genomic alteration in each study. (C) Boxplot showing the distribution of mutation counts corresponding to different Copy Number Alteration (CNA) types (e.g., amplification, gain, diploid, shallow deletion) for the NEU1 gene in the MSK-IMPACT cohort. [file Image7.jpeg]

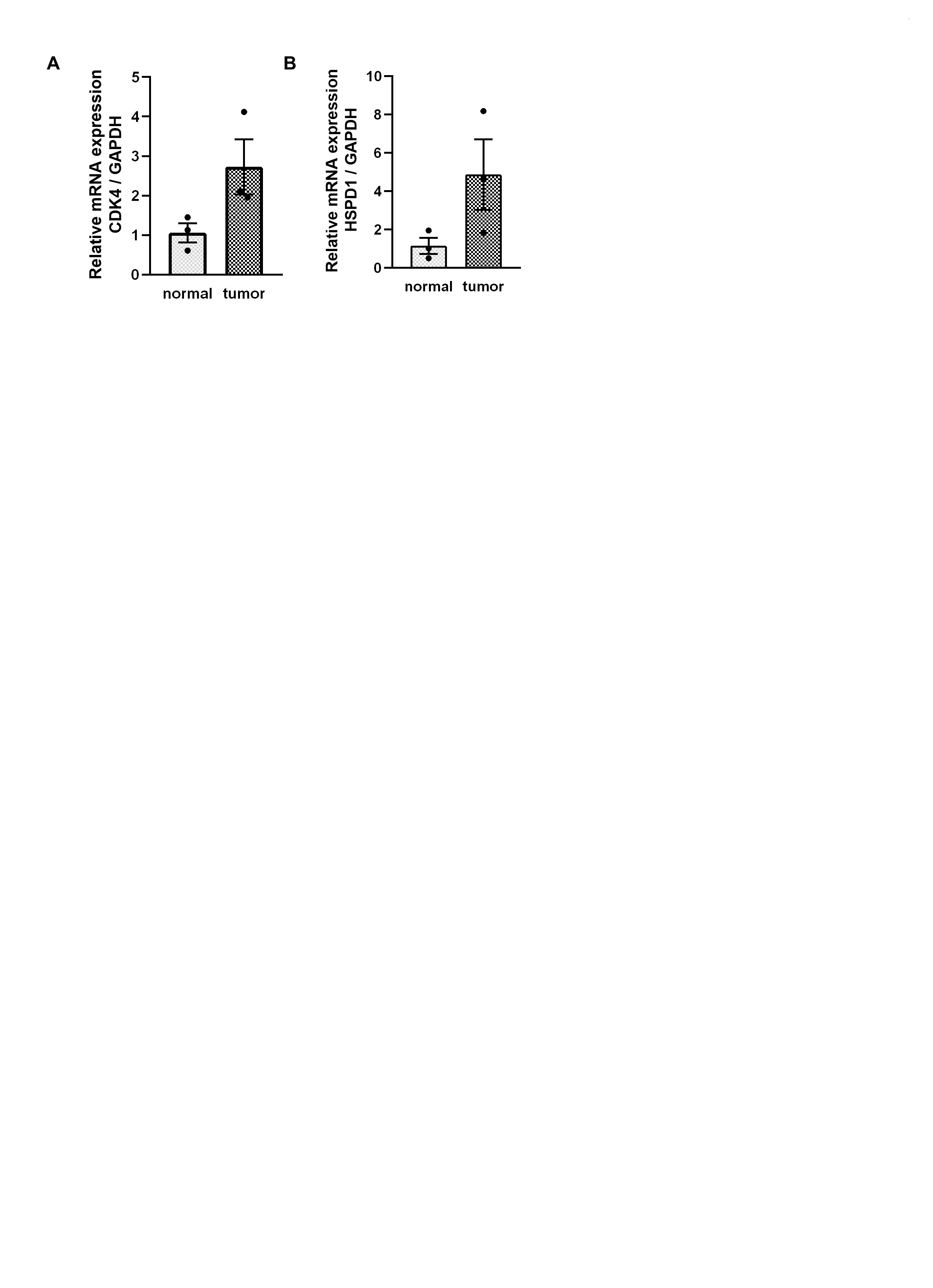

Supplement: Supplementary Figure 8 — Clinical sample validation of CDK4 and HSDP1 mRNA expression levels. [file Image8.jpg]
